# Organic amendment additions to rangelands: A meta‐analysis of multiple ecosystem outcomes

**DOI:** 10.1111/gcb.14535

**Published:** 2019-01-02

**Authors:** Kelly Gravuer, Sasha Gennet, Heather L. Throop

**Affiliations:** ^1^ Center for Biodiversity Outcomes Arizona State University Tempe Arizona; ^2^ The Nature Conservancy Arlington Virginia; ^3^ The Nature Conservancy San Francisco California; ^4^ School of Earth and Space Exploration Arizona State University Tempe Arizona; ^5^ School of Life Sciences Arizona State University Tempe Arizona

**Keywords:** arid, biodiversity, biosolids, climate change, compost, ecosystem services, grassland, runoff, savanna, soil carbon

## Abstract

Interest in land application of organic amendments—such as biosolids, composts, and manures—is growing due to their potential to increase soil carbon and help mitigate climate change, as well as to support soil health and regenerative agriculture. While organic amendments are predominantly applied to croplands, their application is increasingly proposed on relatively arid rangelands that do not typically receive fertilizers or other inputs, creating unique concerns for outcomes such as native plant diversity and water quality. To maximize environmental benefits and minimize potential harms, we must understand how soil, water, and plant communities respond to particular amendments and site conditions. We conducted a global meta‐analysis of 92 studies in which organic amendments had been added to arid, semiarid, or Mediterranean rangelands. We found that organic amendments, on average, provide some environmental benefits (increased soil carbon, soil water holding capacity, aboveground net primary productivity, and plant tissue nitrogen; decreased runoff quantity), as well as some environmental harms (increased concentrations of soil lead, runoff nitrate, and runoff phosphorus; increased soil CO_2_ emissions). Published data were inadequate to fully assess impacts to native plant communities. In our models, adding higher amounts of amendment benefitted four outcomes and harmed two outcomes, whereas adding amendments with higher nitrogen concentrations benefitted two outcomes and harmed four outcomes. This suggests that trade‐offs among outcomes are inevitable; however, applying low‐N amendments was consistent with both maximizing benefits and minimizing harms. Short study time frames (median 1–2 years), limited geographic scope, and, for some outcomes, few published studies limit longer‐term inferences from these models. Nevertheless, they provide a starting point to develop site‐specific amendment application strategies aimed toward realizing the potential of this practice to contribute to climate change mitigation while minimizing negative impacts on other environmental goals.

## Graphical abstract

On rangelands, application of organic amendments such as compost and biosolids is increasingly proposed for climate change mitigation. Considering a range of ecosystem outcomes, this meta‐analysis found that organic amendments, on average, provide some environmental benefits (increased soil carbon, soil water holding capacity, aboveground net primary productivity, and plant tissue nitrogen; decreased runoff quantity), as well as some environmental harms (increased concentrations of soil lead, runoff nitrate, and runoff phosphorus; increased soil CO_2_ emissions). Our models can be used to design site‐specific amendment application strategies, helping to realize the potential benefits of this practice while minimizing environmental harms.

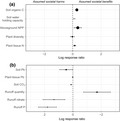

## 1 INTRODUCTION

Organic amendments—materials of plant or animal origin that can be added to soil, such as manures, biosolids, green wastes, and composts—are applied to millions of acres of mesic or irrigated croplands each year, where they can boost soil carbon (C) and fertility (Diacono & Montemurro, 2010; Hargreaves, Adl, & Warman, 2008; Khaleel, Reddy, & Overcash, 1981; National Research Council, 2002). There is currently a resurgence in interest and funding for soil health practices including organic amendment applications because of their on‐site and public benefits, including contribution to climate change mitigation (Paustian et al., 2016). Organic amendments have also been applied to rangelands, often to boost plant productivity in areas that have been heavily grazed or eroded (Fresquez, Francis, & Dennis, 1990; Hanke, Gröngröft, Jürgens, & Schmiedel, 2011; Kowaljow, Mazzarino, Satti, & Jiménez‐Rodríguez, 2010), or as an alternative to landfill disposal of the materials (Cabrera et al., 2009; Sullivan, Stromberger, Paschke, & Ippolito, 2006). Interest in organic amendment application to rangelands has been increasing in parallel to soil health efforts in croplands, bolstered by growing interest in using these materials to restore degraded rangelands, a need that may expand as global changes increasingly challenge these lands (Huang et al., 2017). However, practices seeking to increase soil C might be less effective on untilled rangelands than on croplands, pastures, or restoration sites such as abandoned mines, since prior soil disturbance of most cropland, pasture, or restoration sites would decrease soil C pools, enabling greater proportional increases in soil C pools following amendments (Lal, 2018; Larney & Angers, 2012). In general, the efficacy and outcomes of this practice on rangelands are relatively poorly studied, and the potential for negative environmental consequences are higher in rangelands than croplands due to their starkly different ecology and management context, as detailed below.

Rangelands cover more than 30% of terrestrial lands, and include grasslands, savannas, scrub, and woodlands. Domestic livestock grazing is the primary, and often only, viable economic use of rangelands; across their vast area, these lands support the livelihoods of approximately 1 billion people (Sayre, McAllister, Bestelmeyer, Moritz, & Turner, 2013). Unlike crop or pasture‐based systems, rangeland management does not typically include tillage, irrigation, nutrient inputs, or other intensive practices. Instead, rangelands are typically managed as natural or seminatural ecosystems, in which managers are often, though not always (e.g., Bedunah & Angerer, 2012), able to manage livestock not only for beef production, but also for ecological outcomes such as invasive plant control, fire fuels reduction, protected species habitat, and, more recently, soil C storage. Collectively, these rangeland ecosystem services benefit billions more people globally (Havstad et al., 2007; Sayre et al., 2013). In many regions, including biomes such as temperate grasslands and Mediterranean ecosystems where biodiversity is most threatened (Hoekstra, Boucher, Ricketts, & Roberts, 2005), rangelands are an important biodiversity stronghold (e.g., Cameron, Marty, & Holland, 2014). Many rangeland managers recognize the multifunctional nature of these landscapes, and will often, though not always (e.g., Cáceres, Tapella, Quétier, & Díaz, 2015), consider the impacts of new management practices—such as organic amendment application—on both the economic and ecological sustainability of their operations (Roche et al., 2015).

Most rangelands today are found in arid, semiarid, or Mediterranean climates, as mesic nonforested lands tend to be converted to croplands or pastures with higher economic returns (Sayre, 2017). In these dry climates, temperature and precipitation patterns—which are highly variable within and between years—exert stronger controls on vegetation productivity and composition than do human management actions (Booker, Huntsinger, Bartolome, Sayre, & Stewart, 2013; Westoby, Walker, & Noy‐Meir, 1989). This has two implications for the outcomes of management practices such as organic amendment additions. First, if application of a practice does not happen to coincide with climatic conditions that enable the system to respond to it, the practice may not have the intended impact (Brown & Herrick, 2016; Derner et al., 2018; Walton, Herrick, Gibbens, & Remmenga, 2001). For example, if a series of dry years followed amendment application, opportunities for amendment nutrients to stimulate plant growth may be limited. Second, if a management practice does lead to a change in the system state but the manager determines the change to be undesirable, climatic patterns may constrain the manager's ability to reverse the change (Friedel, 1991; Westoby et al., 1989). For example, if amendment application promoted a fire‐prone invasive plant, subsequent dry years that encouraged fires might reinforce the dominance of the undesirable species, regardless of management efforts. The typically low revenue per acre on rangelands (Huntsinger, Bartolome, & D'Antonio, 2007) may reinforce this constraint, by limiting capital available to invest in intensive management practices such as herbicides to remove undesirable vegetation.

Despite the possibility that organic amendments are less beneficial in dry rangelands than in more mesic systems, studies to date have shown that organic amendment applications to rangelands can provide certain benefits, including boosting forage quantity and quality for livestock (e.g., Martínez, Cuevas, Calvo, & Walter, 2003; Sullivan et al., 2006; Jurado‐Guerra, Luna‐Luna, Flores‐Ancira, & Saucedo‐Teran, 2013), reducing soil erosion (e.g., Ros, Garcia, & Hernandez, 2001; Crohn, Chaganti, & Reddy, 2013), increasing activity and biomass of soil microbial communities (e.g., Tarrasón, Ojeda, Ortiz, & Alcañiz, 2010; Torres, Bastida, Hernández, Albaladejo, & García, 2015), and improving water retention (e.g., Albaladejo, Castillo, & Díaz, 2000; Rostagno & Sosebee, 2001). Recent work has also highlighted the potential for increased C storage in Mediterranean‐climate rangelands following amendment application (Ryals, Hartman, Parton, DeLonge, & Silver, 2015; Ryals, Kaiser, Torn, Berhe, & Silver, 2014; Ryals & Silver, 2013), which could provide climate change mitigation (DeLonge, Ryals, & Silver, 2013). Considering the enormous area of rangelands, these results have spurred the inclusion of organic amendment application to rangelands as a strategy in large‐scale assessments of how natural and working lands can contribute to mitigating climate change (Cameron, Marvin, Remucal, & Passero, 2017).

Although organic amendment applications to rangelands may provide benefits, applications can also pose risks to public health or the environment. For example, some amendments contain heavy metals such as nickel, lead, and cadmium, which may leach into soils and water supplies and accumulate in plants, animals, and humans (Goss, Tubeileh, & Goorahoo, 2013; Manzetti & van der Spoel, 2015). Amendment analyses prior to application can help to minimize this risk, and some governments have regulated heavy metal content of some types of land‐applied amendments (Hill, 2005; United States Environmental Protection Agency, 1993). In addition, nutrients present in amendments, particularly nitrate and phosphate, may be transported to ground and surface waters (e.g., Aguilar & Loftin, 1992; Stout, Weaver, Gburek, Folmar, & Schnabel, 2000; Tejada & Gonzalez, 2008), where they can negatively impact aquatic life and water quality for human use.

The probability of negative impacts increases if risks are not managed by matching application sites with appropriate amendment compositions and application strategies. For example, nutrients in organic amendments may enhance plant‐available nutrients, the risks of which are particularly great in naturally nutrient‐poor systems like many rangelands. Nutrient additions in these systems can favor fast‐growing, often nonnative species at the expense of native biodiversity (e.g., Borer et al., 2014; Gea‐Izquierdo, Gennet, & Bartolome, 2007; González et al., 2010; Harpole et al., 2016; Seabloom et al., 2015; Stevens, Dise, Mountford, & Gowing, 2004; Suding et al., 2005). These effects have been observed even with relatively low nutrient inputs (Clark & Tilman, 2008) and even when nutrients are primarily in organically bound forms (Bastida et al., 2008; Blumenthal, Lecain, & Augustine, 2017; Stavast et al., 2005). Furthermore, over time, decreases in plant diversity or increases in invasive species could potentially reduce primary productivity (Isbell et al., 2013) and/or the seasonal availability and quality of forage (Haferkamp, Grings, Heitschmidt, MacNeil, & Karl, 2001). Altogether, these considerations suggest that analysis of the potential benefits and risks of organic amendment addition to rangelands—including which types of amendments and sites are likely to maximize benefits and minimize harms—could provide a valuable basis for decision‐making in light of increasing interest in this practice.

Here we present a meta‐analysis of organic amendment additions to rangelands in arid, semiarid, and Mediterranean climates, synthesizing amendment effects on a suite of ecosystem outcomes. Specifically, we analyzed 11 ecosystem outcomes that had sufficient published data: soil organic C concentration, soil water holding capacity, aboveground net primary productivity (ANPP), plant species diversity, plant tissue nitrogen (N) concentration, soil CO_2_ emissions, soil lead (Pb) concentration, plant tissue Pb concentration, runoff quantity, runoff P, and runoff nitrate (Table 1). We also analyzed nine additional variables (e.g., soil N, cover of annual vs. perennial plants; hereafter “supporting variables”) that could inform hypothesized mechanisms for outcomes’ responses. After modeling how effect sizes for the eight most data‐rich outcomes varied according to climate zone, time since application, amount of amendment applied, and amendment N concentration, we used those models to compare benefits and harms for defined amounts of amendment applied and amendment N concentrations.

**Table 1:** Sample size for calculation of effect sizes representing the impact of organic amendment addition on a variety of rangeland ecosystem properties (response variables). Response variables were designated as “outcomes” if they were indicative of ecosystem services whose importance to society had been demonstrated (Table 3). Response variables that were less directly indicative of such ecosystem services, but still potential contributors to mechanisms underlying outcome responses, were designated as “supporting variables.” “Experiment” refers to a specific set of field plots; publications in which the same treatments were applied at more than one site were considered to contain more than one experiment. “Observation” was defined as a unique combination of response variable + experiment + measurement date + amendment type + amount of amendment applied. All outcomes and supporting variables listed here were considered to have sufficient data for effect size estimation, which was defined as 10 or more observations from 3 or more experiments. Explanatory models were built for outcomes only (not for supporting variables), and an outcome was considered to have sufficient data for explanatory model construction if 50 or more observations from 5 or more experiments were available. Data for the following outcomes were also sought, but insufficient observations were found to estimate effect sizes: cover of exotic (vs. native) plants (1 observation from 1 experiment found), soil N_2_O emissions field measurement (9 observations from 2 experiments found), and soil CH_4_ emissions field measurement (9 observations from 2 experiments found)

	Response variables	Number of experiments	Number of publications	Number of observations	Sufficient data for explanatory models?
Outcomes	Soil Organic C	27	37	244	yes
	Soil Water Holding Capacity	6	5	25	no
	Soil Pb	7	13	88	yes
	Soil CO_2_ Emission (Field)	4	3	41	no
	Aboveground NPP	28	38	271	yes
	Plant Species Diversity	13	13	129	yes
	Plant Tissue N	11	16	128	yes
	Plant Tissue Pb	4	6	43	no
	Runoff Quantity	14	12	85	yes
	Runoff Nitrate	9	8	57	yes
	Runoff P	9	8	72	yes
Supporting Variables	Soil Moisture	9	10	129	NA
	Soil Respiration (Laboratory)	9	15	119	NA
	Soil Microbial Biomass or Abundance	17	19	142	NA
	Soil Total N	22	31	166	NA
	Soil Ammonium (NH_4_^+^)	8	14	122	NA
	Soil Nitrate (NO_3_^‐^)	10	17	143	NA
	Soil Extractable P	18	24	175	NA
	% Cover of Annual Plants	3	3	14	NA
	% Cover of Grass Plants	5	5	53	NA

## 2 MATERIALS AND METHODS

### 2.1 Selection of papers

We systematically searched for published field studies that reported effects of organic amendments on one or more ecosystem properties. We first performed a Web of Science search and then identified additional studies by screening cited references in papers obtained. Gray literature cited by a peer‐reviewed paper was considered eligible if it met all other criteria.

We used the following criteria to identify eligible studies: (a) field experiment with control (no amendment) and treatment (amendment) plots; (b) nonforested ecosystem with sufficient plant biomass to support livestock grazing; (c) carbon‐based amendment to which synthetic nutrients had not been added; (d) soil not subjected to “severe disturbance” within the 20‐year period prior to experiment initiation (as reported by the paper's authors); we considered severe disturbance to include activities such as mining, roadcuts, and agricultural tillage; (e) arid, semiarid, or Mediterranean Köppen–Geiger climate zone (zone B or Cs, as defined by Peel, Finlayson, & McMahon, 2007); and (f) reported effect of amendment addition on at least one of 20 prospective response variables (Table 1). In our response variables, we included one heavy metal (Pb) rather than a suite of metals in order to keep the total number of response variables manageable; Pb was selected due to its impacts on human and livestock health (Table 3). Response variables were designated as “outcomes” if they were indicative of ecosystem services whose importance to society had been demonstrated in the literature, while response variables that were less directly indicative of such services, but still potential contributors to mechanisms underlying outcome responses, were designated as “supporting variables.”

The general format of the search was (ecosystem descriptor) AND (organic amendment descriptor) AND (climate descriptor) (see Table 2 for specific search terms). In the climate descriptor terms, we included the names of countries containing areas with Mediterranean (Köppen–Geiger Cs) climates, as the descriptors used for this type of climate tended to be less consistent than those used for arid and semiarid (Köppen–Geiger B) climates. Regardless of the search terms they contained, we recorded and mapped the locations of all studies to confirm that they fell within a Köppen–Geiger B (hereafter referred to as “dryland”) or Cs climate. The search was conducted in July 2017 and returned 771 papers.

**Table 2:** Search terms for Web of Science search; the three rows were combined using AND

Ecosystem descriptor	grassland* OR rangeland* OR savanna* OR woodland* OR shrubland* OR desert* OR dryland* OR steppe* OR chaparral* OR prairie* OR scrub*
Organic amendment descriptor	"organic amend*" OR "organic waste*" OR compost* OR vermicompost* OR manure* OR slurry* OR biosolid* OR sewage* OR sludge* OR biochar* OR digestate* OR hydrolysate* OR "solid waste*" OR "green waste*" OR "municipal waste*" OR mulch* OR sawdust
Climate descriptor	desert OR dryland* OR steppe* OR arid* OR semi‐arid* OR semiarid* OR Mediterranean* OR California* OR Australia OR Bosnia OR Herzegovina OR Bulgaria OR Chilé OR Chile OR Croatia OR Cyprus OR Egypt OR France OR Greece OR Iraq OR Israel OR Italy OR Jordan OR Kosovo OR Lebanon OR Libya OR Macedonia OR Malta OR Monaco OR Montenegro OR Morocco OR Palestine OR Portugal OR "San Marino" OR Slovenia OR "South Africa" OR Spain OR Syria OR Tunisia OR Turkey

**Table 3:** Outcomes measured and rationale for whether an increase in their effect size was assumed to be beneficial or harmful to society. Based on evidence and discussions in the literature, we made an assumption about whether the societal consequences of a positive effect size for each outcome would be mostly beneficial or mostly harmful. These assumptions about benefits and harms were used to discuss the results of overall effect size and explanatory models (Figure 1, Table 4) and predicted outcomes for a set of amount of amendment applied + amendment N concentration scenarios (Figure 4, Supporting Information Table S5)

Outcome	Assumption about societal consequences of positive effect size	Rationale
Soil organic C concentration	Beneficial	Improves soil structure, infiltration, and water holding capacity, which reduces runoff and erosion and helps to stabilize downstream water supplies (Fynn et al., 2009; Herrick & Wander, 1998). Mitigates climate change by storing carbon below ground that could otherwise be released into the atmosphere as CO_2_ (Follett & Reed, 2010; Fynn et al., 2009). Traps and transforms some pollutants, attenuating their environmental impacts (Herrick & Wander, 1998).
Aboveground net primary productivity (ANPP) of herbaceous vegetation or Total vegetation cover	Beneficial	Supports food supply and security (livestock meat) (Yahdjian, Sala, & Havstad, 2015). Reduces runoff and erosion (Ludwig, Wilcox, Breshears, Tongway, & Imeson, 2005; Wolfe & Nickling, 1993). Contributes to climate change mitigation by removing carbon from atmosphere, some of which may be transferred to slow‐cycling soil pools (Garnett et al., 2017).
Plant species diversity (richness, evenness, and combined indices)	Beneficial	More diverse plant communities tend to be more productive and to have more stable productivity over time, resisting the declines in productivity with extreme climatic events that can befall less diverse plant communities (Isbell et al., 2015; Tilman, Reich, & Knops, 2006). This greater stability supports food security. Also, many people believe that species have value in and of themselves (intrinsic value) (Vucetich, Bruskotter, & Nelson, 2015).
Plant tissue N concentration	Beneficial	Protein is an essential nutrient for livestock nutrition, and crude protein concentration is strongly correlated with nitrogen concentration (National Academies of Sciences, Engineering, & Medicine, 2016). Better livestock nutrition supports food security.
Soil water holding capacity	Beneficial	Improves stability of plant production, particularly in the face of drought (Duniway, Herrick, & Monger, 2010), contributing to food security. Greater capacity for storage of incoming precipitation reduces flood risk (Anderson, 1993).
Soil Pb concentration	Harmful	High levels of lead in soil can inhibit plant growth (US Environmental Protection Agency, 2005), affecting livestock forage base and food security. Direct exposure to lead particles from soil can be detrimental to human and animal health (de Vries, Römkens, & Schütze, 2007; US Environmental Protection Agency, 2005; Xintaras, 1992).
Plant tissue Pb concentration	Harmful	Lead in plants can bioaccumulate in livestock and in humans who consume those livestock, with detrimental health impacts for both (de Vries et al., 2007).
Soil CO_2_ emissions (field measurement)	Harmful	CO_2_ emissions contribute to climate change, which has detrimental impacts on society (Intergovernmental Panel on Climate Change, 2014).
Runoff quantity	Harmful	Runoff increases erosion, which reduces rangeland soil fertility (with implications for long‐term productivity and food security) as well as surface water quality (Bartley et al., 2006; Pimentel, 2006). Runoff also decreases soil water storage, decreasing stability of plant production and increasing flood risk (Wilcox, Maitre, Jobbagy, Wang, & Breshears, 2017).
Runoff P	Harmful	P in runoff can stimulate excessive growth of algae and aquatic plants in surface waters, causing eutrophication that can harm aquatic wildlife and fisheries, restrict water use for recreation, reduce drinking water quality, and promote blooms of certain algal species that have negative human health consequences (Sharpley, McDowell, & Kleinman, 2001).
Runoff nitrate	Harmful	In some waters—especially estuarine, coastal, and marine systems—algal growth is limited by N or is colimited by N and P, such that additional N can be the key factor leading to eutrophication and associated impacts described above (Conley et al., 2009). High levels of nitrate in drinking water can cause methemoglobinemia (“blue baby syndrome”) and potentially other human health impacts (Ward et al., 2005).

### 2.2 Data extraction

Multiple papers reporting different response variables or measurement dates were often published about the same set of field plots (“experiment”), and we assigned each observation to an experiment (in addition to recording its source publication) so that we could account for this nonindependence in subsequent analyses. In our data set, an “observation” was defined as a unique combination of response variable + experiment + measurement date + amendment type + amount of amendment applied. For each observation, we extracted response variable means and standard deviations, the numbers of replicate plots, and *p*‐values or other metrics of statistical difference for treatment vs. control comparisons. When these data were solely displayed in figures, we used WebPlotDigitizer (Rohatgi, 2017) to extract them.

We also extracted data from each paper that could potentially explain variation in outcome effect sizes across studies. These data included amendment properties (moisture, organic C, total N, C:N, ammonium, nitrate, total P, extractable P, total K, extractable K, total Fe), site properties (latitude, longitude, climate zone, MAT, MAP, plant community, management during study, and soil properties [soil type, organic C, total N, extractable P, extractable K, total Fe, pH, texture, bulk density]), and implementation details (date of amendment application, date of measurement, amendment description, amount of amendment applied, soil sample depth).

### 2.3 Data analysis

All analyses were conducted in R (R Core Team, 2018). To avoid multicollinearity and loss of data, we sought to narrow potential predictors to a smaller set of variables that were (a) reported by most studies, (b) not highly correlated, and (c) representative of different categories of variation among studies. Ultimately, we chose five predictors—climate zone, days between amendment application and measurement, amount of amendment applied, amendment total N concentration, and (for soil outcomes) soil sample depth—that were reported for 91%–100% of observations, had relatively low intercorrelations (|*r*| = 0.016–0.209), and included an amendment property, a site property, and key implementation details. Some of these were strongly correlated with other variables in the same category, suggesting they were at least somewhat representative. For example, amendment total N concentration was correlated with amendment total phosphorus (P) concentration (*r* = 0.504) and climate zone was a strong predictor of soil organic C and total N at the application site (*t* = −17.33 and −18.49, respectively, both *p* < 0.0001). We considered using a continuous aridity index calculated from MAT and MAP (Quan, Han, Utescher, Zhang, & Liu, 2013) instead of the categorical climate zone, but since the distribution of the continuous predictor was strongly bimodal with peaks corresponding to the two climate zones, we considered the categorical variable a better representation.

Effect sizes were modeled as log response ratios, with variances calculated from means, standard deviations, and replicates as in Hedges, Gurevitch, and Curtis (1999), without assuming equal variances within treatment and control groups. If neither standard deviations nor *SE*s were reported but a *p*‐value was provided for the control vs. treatment comparison, we calculated the *z*‐score for that *p*‐value and then calculated variance from the *z*‐score and log response ratio using confidence interval formulas in Hedges et al. (1999). If the statistical significance of the control vs. treatment comparison was reported as a *p*‐value range (e.g., *p* < 0.05 or 0.001 < *p* < 0.01), we estimated the *p*‐value as the midpoint of the reported range (e.g., 0.025 for *p* < 0.05) and then used this estimate to calculate variance as above.

We then used the log response ratios and their variances to estimate the overall effect size for each outcome and supporting variable using a random effects model implemented with the rma.mv function in the metafor package (Viechtbauer, 2010). An outcome or supporting variable was considered to have sufficient data for effect size estimation if 10 or more observations from 3 or more experiments were available. Because many of the studies reported significance as *p*‐value ranges rather than providing standard deviations, and some of the effect size variances were therefore estimated, we chose not to weight the effect sizes by the inverse of variance in these models. All models included experiment and publication within experiment as random effects to reflect nonindependence of observations made on the same plots and/or in the same study. Models for some response variables included an additional random effect to account for differences in measurement methods. Specifically, random effects were included to denote whether soil Pb was measured as extractable or total; whether ANPP was measured as mass or cover; whether plant species diversity was measured as richness, evenness, or an integrated index; whether quantity of soil microbes was measured as biomass or abundance; and whether soil moisture was measured volumetrically or gravimetrically.

For each outcome, we next explored which (if any) of our five predictor variables explained important variation in effect sizes among studies. An outcome was considered to have sufficient data for explanatory model construction if 50 or more observations from 5 or more experiments were available. For some outcomes, few studies had been conducted in Mediterranean climates (i.e., the majority of observations were from dryland climates) and there were thus insufficient data to estimate the importance of climate zone with reasonable accuracy; as such, climate zone was included as a (fixed) predictor only if at least three experiments from each climate zone were available (Supporting Information Table S1). Continuous predictors were log‐transformed, centered, and scaled prior to analysis. For each outcome, models with all possible combinations of predictors were built using the rma.mv function in the metafor package (Viechtbauer, 2010) within the glmulti function in the glmulti package (Calcagno, 2013), and model‐averaged coefficient estimates were calculated as weighted averages of coefficient estimates from all such models, using model probabilities as weights. Two methods were used to differentiate important from nonessential predictors. First, predictors were considered important if they had a model‐averaged importance ≥0.8, a commonly used cutoff (Everaert, Deschutter, De Troch, Janssen, & De Schamphelaere, 2018; Terrer, Vicca, Hungate, Phillips, & Prentice, 2016; Whittingham et al., 2009). Second, predictors were considered important if their estimated 95% confidence intervals did not overlap 0; these intervals were calculated taking two sources of uncertainty into account (uncertainty within a given model and uncertainty as to which model is “best”).

For these explanatory models, we ran models with main effects only as well as models with all two‐way interactions among predictors (except soil sample depth, which we viewed as a covariate and included only as a main effect). As many of the data sets had insufficient power to reliably model higher order interactions, we chose to include only two‐way interactions in all models to allow straightforward comparisons.

### 2.4 “Equal inputs” analysis

Because our overall effect sizes were based on subsets of studies with different characteristics (e.g., different median amounts of amendment applied; Supporting Information Table S1), they represent the balance of outcomes recorded from studies conducted to date, not the balance of outcomes that might result from a consistent amendment application strategy. As this latter balance is also of interest, we produced predictions from our model sets for a standard set of hypothetical scenarios with set values for all predictor variables. Because amendment application rate and amendment N concentration were the predictors with highest importance in the explanatory models, we constructed scenarios including ranges of values for these two variables. Value ranges (10–50 Mg/ha total amount applied of amendments with 1.2%–3.6% N) for these scenarios were chosen to be relevant to current incentive programs for organic amendment applications on US rangelands (e.g., State of California, 2016), which have focused predominantly on incentivizing compost application at 9–70 Mg/ha (e.g., Haden, Gryze, & Nelson, 2014, Gravuer & Gunasekara, 2016). For each outcome, predictions were generated using the multimodel set with lowest AIC value (main effects only or with all two‐way interactions). To generate the predictions, time between amendment application and measurement was set to three years in order to model time frames that were well within the range of empirical data (Supporting Information Table S1). Climate—coded in the models as a dummy variable with dryland = 0 and Mediterranean = 1—was set to 0.5.

Once we had generated predictions for the eight outcomes under all 25 scenarios (combinations of 5 amendment application rates and 5 amendment N concentrations), we illustrated one possible system for determining which of the scenarios “maximized benefits” and “minimized harms” (according to our assumptions about societal consequences for each outcome; Table 3). To do this, we counted how many of the eight outcomes had positive vs. negative predicted effect sizes, and how many had effect size confidence intervals that did vs. did not overlap zero. The direction of the effect sizes whose confidence intervals did not overlap zero was the primary criterion, and the direction of the effect sizes whose confidence intervals did overlap zero was used to further distinguish among scenarios that had the same result for the first criterion. Importantly, our concept of “benefits” and “harms” here considers only the direction of the effect sizes; it does not consider effect size magnitudes relative to external benefit or harm definitions (e.g., regulatory standards, ranchers’ perceptions of meaningful changes) because such definitions are likely to vary greatly across contexts.

## 3 RESULTS

### 3.1 Overall effect sizes

**Figure 1:**
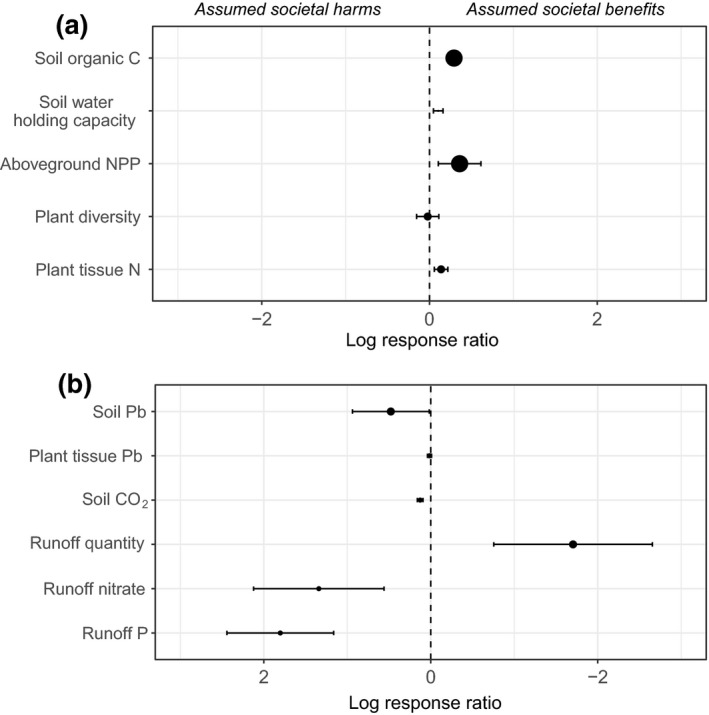
Overall effect sizes (log response ratios) for the effect of organic amendment addition on rangeland ecosystem outcomes, based on a meta‐analysis of published studies. Point sizes are proportional to the number of observations upon which each effect size is based (Table 1). The *x*‐axis is inverted in (b) relative to (a) so that assumed societal benefits are on the right side of the figure and assumed societal harms are on the left side of the figure in both cases. The *x*‐axis shows the natural log of the response ratio, which was used in all quantitative analyses, whereas Results text describes raw response ratios, which have more intuitive application. Because data on each outcome were reported by different subsets of studies (Supporting Information Table S4), these effect sizes should not be additively compared (e.g., soil organic C and ANPP cannot be directly compared to soil CO_2_ emissions)

For the set of studies meeting our criteria for each outcome (Table 1), amendment addition increased soil organic C concentration (mean = 1.34×), soil water holding capacity (mean = 1.11×), ANPP (mean = 1.43×), and plant tissue N concentration (mean = 1.15×) (Figure 1a). Impacts on plant species diversity were essentially neutral (mean = 0.98×, confidence interval includes 1; Figure 1a). Amendment addition also decreased runoff quantity (mean = 0.18×) while increasing runoff nitrate concentration (mean = 3.82×), runoff P concentration (mean = 6.07×), soil Pb concentration (mean = 1.61×), and soil CO_2_ emissions (mean = 1.14×) (Figure 1b). Impacts on plant tissue Pb concentration were essentially neutral (mean = 1.01×, confidence interval includes 1; Figure 1b). Mean values of outcomes in control (unamended) and treatment (amended) plots are in Supporting Information Table S2.

**Figure 2:**
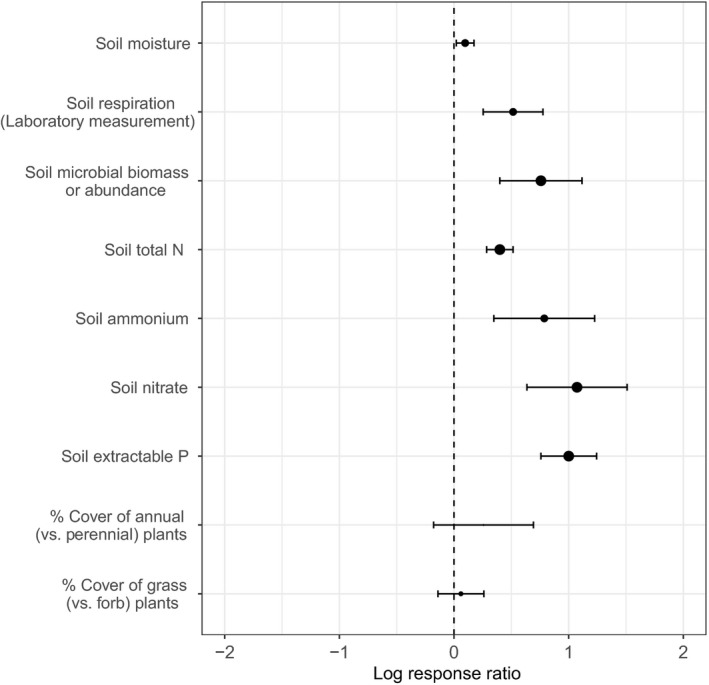
Overall effect sizes (log response ratios) for the effect of organic amendment addition on supporting variables, based on a meta‐analysis of published rangeland studies. Point sizes are proportional to the number of observations upon which each effect size is based. As in Figure 1, the x‐axis shows the natural log of the response ratio, whereas Results text describes raw response ratios, which have more intuitive application. Again, because data on each variable were reported by different subsets of studies (Supporting Information Table S4), these effect sizes should not be additively compared (e.g., soil respiration cannot be directly compared to soil microbial biomass)

For supporting variables, amendment addition increased values of a variety of soil properties, including moisture (mean = 1.10×), laboratory‐measured respiration (mean = 1.67×), microbial biomass or abundance (mean = 2.13×), total N (mean = 1.49×), ammonium (mean = 2.20×), nitrate (mean = 2.92×), and extractable P (mean = 2.72×) (Figure 2). Few studies reported sufficient statistical information for relative cover of grasses (vs. forbs) or relative cover of annuals (vs. perennials) to allow inclusion in effect size calculation. With consequently limited power, confidence intervals for both of these effect sizes included zero, although there was a trend toward increased percentage of annuals (mean = 1.29× [95% CI = 0.84–2.00]; Figure 2).

### 3.2 Explanatory models

The five predictors (climate zone, days between amendment application and measurement, amendment application rate, amendment total N concentration, and [for soil outcomes] soil sample depth) together explained significant heterogeneity among effect sizes in all eight outcome models (all tests of moderators *p* ≤ 0.01; Supporting Information Table S3).

In the models with main effects only (Table 4A), amount of amendment applied and amendment N concentration made important contributions for more than half of the outcomes. Adding more amendment had (assumed) benefits for four outcomes (soil organic C, ANPP, plant tissue N, runoff quantity) and (assumed) harms for two outcomes (runoff nitrate and runoff P). Adding an amendment with higher N concentration had (assumed) benefits for two outcomes (soil Pb and runoff quantity) and (assumed) harms for four outcomes (soil organic C, runoff nitrate, runoff P, and plant species diversity). Time between amendment application and measurement made an important contribution for two outcomes: soil organic C effect size decreased with time and ANPP effect size increased with time. Climate zone and soil depth did not have high importance for any model in which they were tested.

**Table 4:**
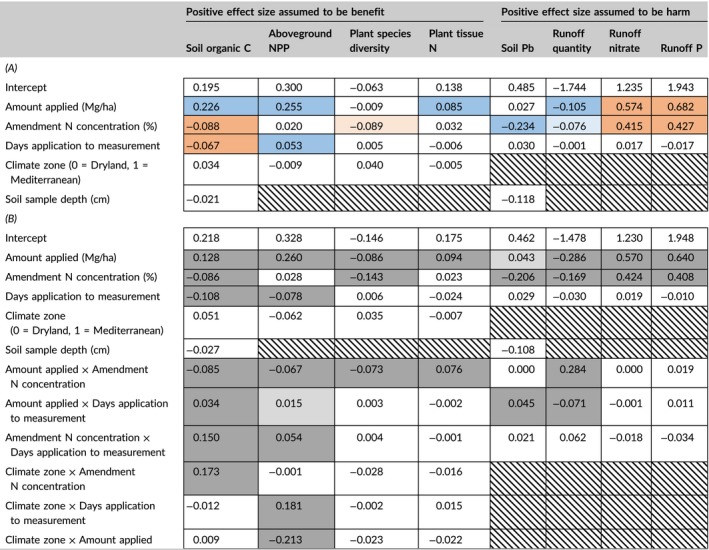
Multimodel‐averaged coefficients from explanatory models. Hatching indicates that a predictor was not included in models for an outcome. Continuous fixed predictors were log‐transformed, centered, and scaled prior to analysis. For each outcome, two methods were used to differentiate important from nonessential predictors. First, predictors were considered important if they had a model‐averaged importance ≥0.8, a commonly used cutoff (Everaert et al., 2018; Terrer et al., 2016; Whittingham et al., 2009). Second, predictors were considered important if their estimated 95% confidence intervals did not overlap 0; these intervals were calculated taking two sources of uncertainty into account (uncertainty within a given model and uncertainty as to which model is “best”) and were more conservative. In the tables below, darker shading indicates that a predictor was important according to both metrics, whereas lighter shading indicates that a predictor was important according to the importance ≥0.8 metric only. (A) Models with main effects only. For outcomes for which a positive effect size was an assumed benefit to society, important predictors with positive coefficients are shaded in blue and important predictors with negative coefficients are shaded in orange. For outcomes for which a positive effect size was an assumed harm to society, important predictors with positive coefficients are shaded in orange and important predictors with negative coefficients are shaded in blue. Thus, the overall benefits and harms of a particular predictor are indicated by its shadings: for example, increasing the amount of amendment applied is estimated to contribute to benefits for four outcomes (soil organic C, aboveground NPP, plant tissue N, and runoff quantity), to contribute to harms for two outcomes (runoff nitrate and runoff P), and to make less important contributions for two outcomes (plant species diversity and soil Pb). (B) Models with all two‐way interactions. Here, predictors important according to the “importance ≥0.8” metric only are indicated with light gray shading and predictors important according to both that metric and the “estimated 95% confidence interval excluding 0” metric are indicated with dark gray shading. No assumptions about benefits or harms are indicated for these relationships, as the inclusion of interactions complicates the interpretation of a positive or negative coefficient [Colour table can be viewed at http://www.wileyonlinelibrary.com]

**Figure 3:**
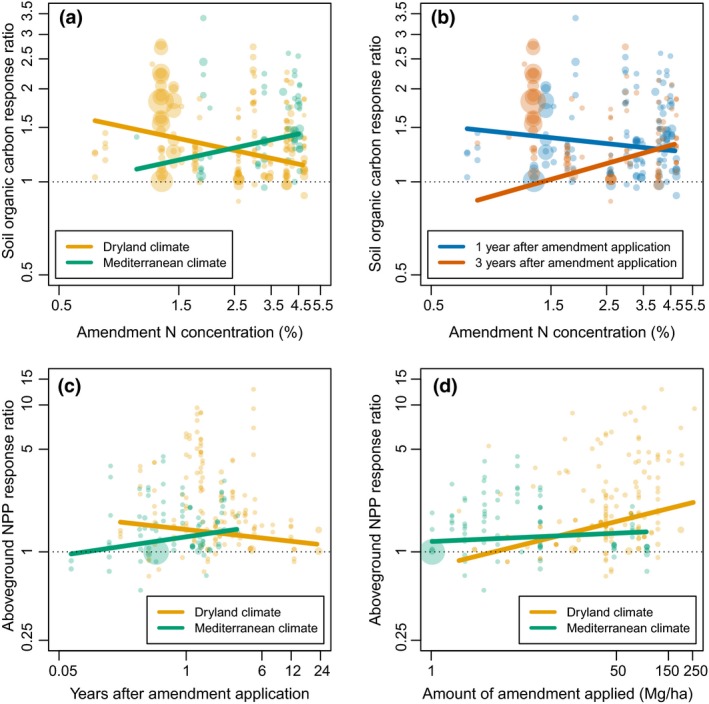
High‐importance interactions in explanatory models for soil organic C and aboveground net primary productivity (ANPP) effect sizes: (a) amendment N concentration × climate zone vs. soil C response ratio (importance = 0.9997); (b) amendment N concentration × time vs. soil C response ratio (importance = 1); (c) time × climate zone vs. ANPP response ratio (importance = 1); and (d) amount of amendment applied × climate zone vs. ANPP response ratio (importance = 1). Note log–log axes in all figures. Points represent observations (unique experiment + measurement date + amendment type + amount applied combinations) and are proportional to the inverse of effect size standard errors, such that larger points indicate more precise observations. In (a), (c), and (d), points are color‐coded by climate zone. In (b), points are color‐coded by time between amendment application and measurement, with blue indicating measurement ≤2 years and red indicating measurement >2 years after application. Lines represent model predictions, with all other variables in the model set to their means

When two‐way interactions were included (Table 4B), many interactions made important contributions. For soil organic C, four of six interactions made important contributions, and the interactions with largest (standardized) coefficients were climate zone × amendment N concentration and days application to measurement × amendment N concentration. Soil organic C tended to increase with amendment N concentration in Mediterranean climates and when measurements were taken longer after application (e.g., 3 years), whereas it tended to decrease with amendment N concentration in dryland climates and when measurements were taken sooner after application (e.g., 1 year; Figure 3a,b). For ANPP, five of six interactions made important contributions, and the interactions with largest (standardized) coefficients were climate zone × days application to measurement and climate zone × amount of amendment applied. In dryland climates, the ANPP boost provided by the amendment decreased with time and increased with the amount of amendment applied, whereas in Mediterranean climates, the boost increased with time but was not greatly affected by amount of amendment applied (Figure 3c,d).

For other outcomes, fewer interactions made important contributions (Table 4B). Interactions with high importance included amount of amendment applied × amendment N concentration for plant species diversity, plant tissue N, and runoff quantity, and amount of amendment applied × days application to measurement for runoff quantity and soil Pb.

Among interactions, amount of amendment applied × amendment N concentration was most frequently important and played out somewhat differently among outcomes (Table 4B, Supporting Information Figure S1). For plant species diversity and plant tissue N, increasing the amount of amendment applied had a stronger effect for high‐N amendments than for low‐N amendments, resulting in a steeper decrease in plant species diversity and a steeper increase in plant tissue N (Supporting Information Figure S1a,b). For soil organic C and runoff quantity, increasing the amount of amendment applied had a stronger effect for low‐N amendments than for high‐N amendments, resulting in a steeper increase in soil organic C and a steeper decrease in runoff quantity (Supporting Information Figure S1c,d).

### 3.3 “Equal inputs” analysis

**Figure 4:**
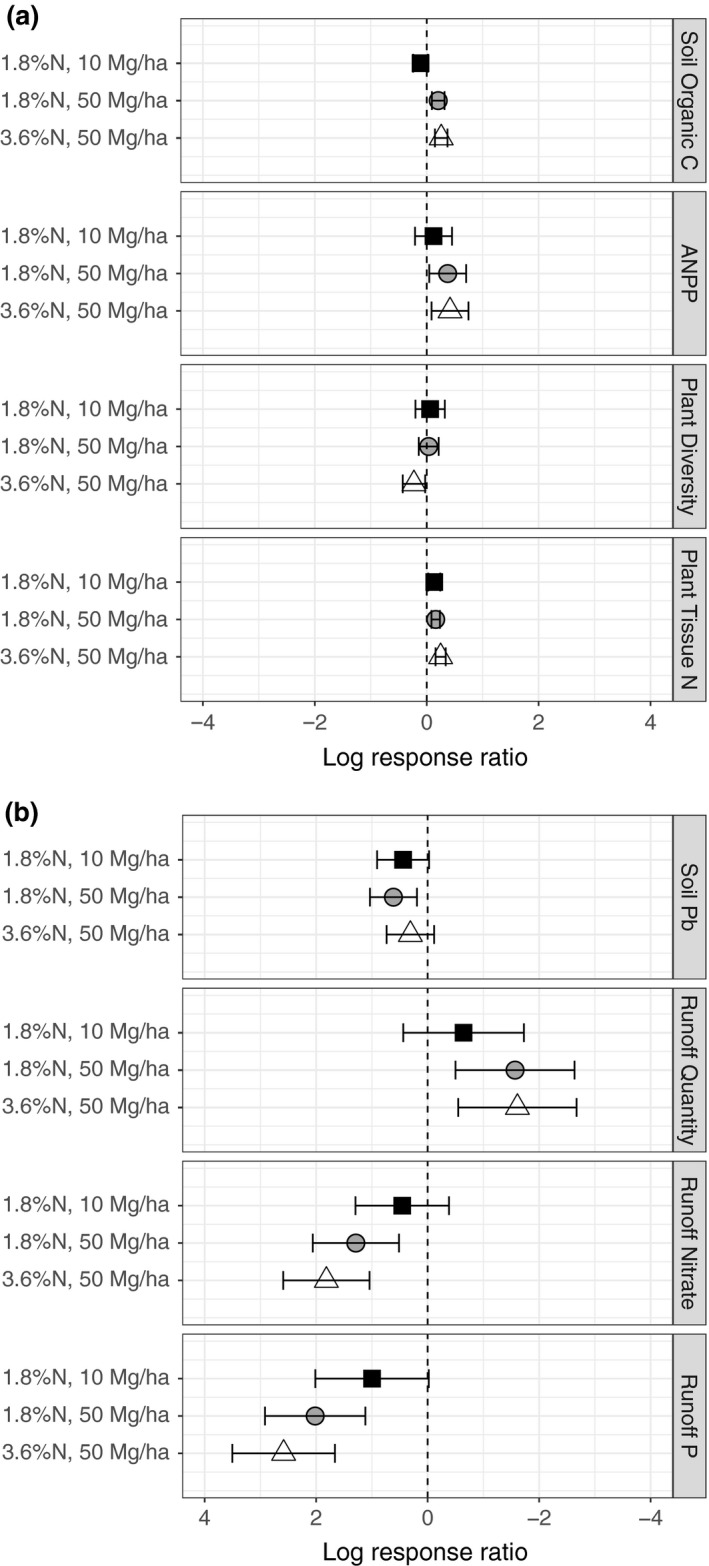
Modeled effect sizes (log response ratios) at 3 years after application for outcomes under three amendment amount + N concentration scenarios. Outcomes for which positive effect size is an assumed societal benefit are shown in (a) and outcomes for which positive effect size is an assumed societal harm are shown in (b). As in Figure 1, the *x*‐axis is inverted in (b) relative to (a) so that assumed societal benefits are on the right side of the figure and assumed societal harms are on the left side of the figure in both cases. The black squares represent the “minimize harms” scenario (10 Mg/ha of 1.8% N amendment), the gray circles represent a “maximize benefits” scenario (50 Mg/ha of 1.8% N amendment), and the white triangles represent a scenario that “maximizes harms” (50 Mg/ha of 3.6% N amendment)

Within the 3‐year modeled time frame, among scenarios we tested, applying relatively high amounts (50 Mg/ha) of relatively low N concentration (1.2%–1.8% N) amendments was predicted to maximize benefits (Supporting Information Table S5). Predicted benefits increased with amount of amendment applied for all amendments. Within scenarios having high application amounts, high amounts of low‐N amendments increased plant species diversity, whereas high amounts of higher N amendments decreased plant species diversity (Supporting Information Table S5, Figure 4, Supporting Information Figure S2).

Among scenarios we tested, applying low amounts (10 Mg/ha) of relatively low N concentration (1.8% N) amendments was predicted to minimize harms (Supporting Information Table S5). As for benefits, predicted harms increased with amount of amendment applied. However, predicted harms had a more complicated relationship with amendment N concentration: runoff nitrate and runoff P increased with amendment N concentration, whereas soil Pb decreased. At low amounts applied, a moderately low‐N (1.8%) amendment was a compromise between these trends, with all three harms being less certain (Supporting Information Table S5).

Overall, a trade‐off between minimizing harms and maximizing benefits was apparent: the scenario that minimized harms also minimized more certain benefits, while the scenarios that maximized benefits also had among the highest numbers of harms. However, keeping amendment N concentration low appeared to avoid the harm to plant species diversity that was likely at high rates of high‐N amendments (Figure 4, Supporting Information Figure S2).

## 4 DISCUSSION

For every outcome we analyzed, the paucity of long‐term studies was a limitation to assessing the long‐term effects of organic amendment application. Follow‐up monitoring of amendment addition plots, as performed by a few studies to date (e.g., Bastida, Hernández, Albaladejo, & García, 2013; Gazol, Uria‐Diez, Elustondo, Garrigó, & Ibáñez, 2016; Ippolito, Barbarick, Paschke, & Brobst, 2010; Sullivan et al., 2006; Torres et al., 2015), could help to fill this data gap. Nevertheless, our analysis revealed patterns that may assist in decision‐making until more long‐term data are available. Specifically, we found that application of organic amendments on rangelands resulted in trade‐offs across environmental outcomes: some outcomes (e.g., plant tissue N) usually benefitted, while other outcomes (e.g., runoff P) were usually harmed. For a few outcomes (e.g., plant species diversity), details of the application scenario—usually the amount of amendment applied and/or its N concentration—affected the probability of benefits versus harms. In particular, at the three‐year postapplication time point we modeled, amendments with relatively low N concentrations (e.g., most composts) were consistent with both maximizing benefits and minimizing harms and thus could be preferable to use of higher N materials (e.g., uncomposted biosolids) in many contexts. However, the amount of amendment applied, regardless of its N concentration, still showed an apparent trade‐off, in which lower application amounts minimized harms but also minimized benefits, and higher application amounts maximized benefits but also increased probability of harms. Although we synthesized data on several C pools and fluxes, the scarcity of studies on net ecosystem C balance was a limitation preventing calculation of effect sizes for this outcome.

### 4.1 Benefits and harms in the “equal inputs” analysis

To compare benefits and harms among modeled scenarios, we chose one of a variety of possible methods: tabulating the direction of modeled effect sizes and whether they statistically differed from zero (Supporting Information Table S5). Importantly, statistically significant effects may or may not be meaningful relative to management goals at any given site. Other criteria may be useful in assessing whether these effects are meaningful for specific goals or contexts. For example, translating ANPP effect size into animal unit months at a site may inform whether the predicted forage productivity boost is meaningful for animal production. At sites where plant community composition is a particular concern, appropriate N critical loads (Fenn et al., 2010; Ochoa‐Hueso et al., 2011; Simkin et al., 2016) may be a useful complement to the plant diversity effect size, as local factors such as soil type will also be important, diversity indices do not capture all plant community changes, and longer‐term effects may differ from those we modeled. Thus, while we have illustrated one way of evaluating the balance of benefits and harms, we emphasize that these results are best considered in reference to the values and goals governing the management of the specific application site.

### 4.2 Climate change mitigation (net ecosystem C balance)

Considering potential benefits of amendment application, we found the largest number of observations for soil organic C and ANPP, with increases in these outcomes consistently reported. Although these are promising indicators of climate change mitigation potential, they do not demonstrate mitigation by themselves. Increases in CO_2_ emissions—from decomposition of the amendment, increased root respiration, and potential consumption of existing soil organic matter—could offset at least some of the plant and soil C gains. Our effect size estimates for soil organic C and ANPP were larger than for field‐based CO_2_ emissions, but because these measurements derived from different subsets of studies, they cannot be directly compared. Furthermore, changes to N_2_O and CH_4_ emissions must also be considered (e.g., Louro, Cárdenas, García, & Báez, 2016, Nichols et al., 2016) and, importantly, the short‐term nature of many published measurements limits understanding of longer‐term mitigation potential. Further studies of how ecosystem greenhouse gas fluxes are affected by amendment addition (e.g., Ryals & Silver, 2013, Ryals et al., 2015), ideally over decades and across a range of sites and conditions, are needed to better understand the climate change mitigation potential of this practice. Additionally, life cycle assessments (e.g., DeLonge et al., 2013) that compare rangeland amendment application to other potential amendment uses (e.g., on croplands, restoration sites, or urban landscapes) would be helpful. Including transportation emissions is important in these comparisons because, on average, transport distances from compost facilities to rangelands may be different than distances to croplands or urban centers. Planning efforts may also benefit from comparing the climate change mitigation potential of this practice to that of other land‐based practices such as changes in forest management (e.g., Cameron et al., 2017).

### 4.3 Soil organic carbon

The increases in soil organic C that we recorded were remarkably consistent: 233 of 244 observations had response ratios >1, and 134 of these observations had a full 95% confidence interval >1. This may seem surprising given the relatively short median time between amendment application and measurement (Supporting Information Table S1); for more than half of observations, <2 years had elapsed, whereas management‐induced soil C increases often take 3 years or more to become apparent (Diacono & Montemurro, 2010; Smith, 2004). Within the methods descriptions of contributing studies, few authors described removing pieces of the amendment itself from soil during sample processing, so at least some of the soil C increase is likely residual amendment material. Furthermore, although a few studies reported an increase in physically protected pools of soil C (Nicolás, Kennedy, Hernández, García, & Six, 2014; Ryals et al., 2014) and some others reported increases in aggregate stability (Ros et al., 2001, Caravaca, Garcia, Hernández, & Roldán, 2002; Caravaca, Figueroa, Alguacil, & Roldán, 2003; Caravaca, Figueroa, Azcón‐Aguilar, Barea, & Roldán, 2003, Bastida, Moreno, García, & Hernández, 2007, Ojeda, Alcañiz, & Bissonnais, 2008, Tejada & Gonzalez, 2008, Wallace et al., 2016; but see Fuentes, Valdecantos, Llovet, Cortina, & Vallejo, 2010, Díaz‐Raviña et al., 2012), most soil organic C measurements did not distinguish between protected and unprotected pools. Additional data distinguishing these different pools of C would better illuminate how likely the increases we documented are to persist over time.

The transfer of amendment and plant C into protected soil C pools is mediated by soil microbial communities. Although we documented an increase in microbial biomass with amendments, suggesting a potential for increase in protected soil C pools, published data were insufficient to calculate effect sizes for other microbial parameters. Studies to date indicate that amendments can decrease fungal to bacterial ratios, increase growth efficiency, and decrease diversity of bacterial and fungal communities (Bastida, Selevsek, Torres, Hernández, & García, 2015; Dennis & Fresquez, 1989; Sullivan, Stromberger, & Paschke, 2006). However, amendment effects on microbial function can vary by amendment and soil type (Bastida et al., 2015; Tarrasón et al., 2010). Further research may illuminate the most promising amendment application strategies for microbially mediated benefits such as protected soil C pools.

In the explanatory models of soil organic C and ANPP, interactions among several predictors had high importance, suggesting that a complex interplay of factors may determine effect sizes. Soil organic C changes reflect changes in plant inputs and processes affecting their stabilization, as well as decomposition of the amendment over time. Other processes could also play a role, potentially including decomposition of existing soil organic matter due to priming from labile fractions of amendment C, which could in theory last for days or weeks following precipitation events (Kuzyakov, 2010; Lai et al., 2013); however, empirical tests suggest priming from amendment C is not common (Cross & Sohi, 2011; Sikora & Yakovchenko, 1995). High‐N amendments would be expected to decompose more quickly because decomposition would be less likely to be N‐limited (Averill & Waring, 2018; Melillo, Aber, & Muratore, 1982; Zhang, Hui, Luo, & Zhou, 2008). High‐N amendments would also be expected to provide more nutrients that could spur plant growth, such that low‐N amendments might provide a greater soil C boost in the shorter term (due to their slower decomposition) while high‐N amendment might provide a greater soil C boost in the longer term (as increased plant inputs are gradually converted into soil C), as was indeed the case in our models (Figure 3b). Finally, although the trend for low‐N amendments suggests that they may provide negligible soil C benefits on longer time scales, we would reiterate the general caveat that because relatively few long‐term observations were available (Table S1), these models have limited power to predict long‐term trends.

### 4.4 Aboveground net primary productivity (ANPP)

Amendment addition can promote plant growth via several mechanisms. First, surface‐applied amendments can act as a mulch that reduces soil moisture loss (Cabrera et al., 2009; Whitford, Aldon, Freckman, Steinberger, & Parker, 1989); mulching effects on plant growth might be greatest where amendment decomposition is slowest and/or where water more significantly limits plant growth. Second, amendment nutrients can gradually enter the soil nutrient pool that is available to plants; available nutrient increases might be greatest where precipitation is high enough to promote nutrient mineralization and transport to plant roots but not so high as to promote nutrient loss via runoff or leaching.

In our analysis of the predominantly short‐term (75% <3 years from application) data available, the ANPP boost provided by the amendment increased over time at Mediterranean sites but decreased over time at dryland sites (Figure 3c); this decrease in ANPP boost over time was also observed in two dryland experiments for which both short‐ and long‐term observations were available (Albaladejo et al., 2000; Bastida et al., 2013; Blumenthal et al., 2017), although some boost was still apparent in most of the measurements made after a decade or more. These differing trends might reflect a relatively greater importance of the mulching effect—presumably highest initially and decreasing gradually over time as the amendment decomposes—at dryland sites, and a relatively greater importance of nutrient provision—which could increase over time as plant nutrients are recycled—at Mediterranean sites, although other explanations are possible. Indeed, we found that soil total N and extractable P increased over time in explanatory models we built for these variables (data not shown). From these ANPP model predictions, it may be tempting to infer that one‐time amendment additions could yield greater long‐term ANPP benefits at Mediterranean sites than at dryland sites, but because relatively few long‐term observations were available, especially from Mediterranean sites, we again caution that these models have limited power to predict long‐term trends. Finally, if plant diversity decreases or invasive species increases were to reduce ANPP (e.g., Isbell et al., 2013), those effects may not be apparent in this data set given the predominance of short‐term observations.

### 4.5 Plant species diversity

Although addition of inorganic N and/or P often reduces plant species diversity (e.g., Harpole et al., 2016; Suding et al., 2005), organic amendment addition did not significantly change plant species diversity—as measured by richness, evenness, and indices combining these two metrics—on average across studies that met our criteria (Figure 1). However, nearly 30% of individual effect sizes showed significant diversity decreases (compared to 5% showing significant increases). Our results may differ from studies of inorganic N and/or P addition because most of the N and P in many organic amendments is in organically bound forms not immediately available to plants. There are exceptions such as manure slurries in which more nutrients are in available forms, but such amendments comprised the minority of our data set. Some organically bound nutrients will be converted to plant‐available forms over time. However, the majority of published observations were made <2 years after amendment application, when amounts of plant‐available N and P released are likely to have been much less than in most inorganic N and P addition studies. Consistent with this reasoning and with previous studies showing higher rates of species loss at higher rates of applied N (e.g., Isbell et al., 2013), the decreases in plant species diversity we found were particularly apparent in studies with relatively large amounts of high‐N amendment applied (Supporting Information Figure S1a), which were also the conditions under which plant tissue N increased most (Supporting Information Figure S1b).

For studies reporting that plant diversity did not change 1 or 2 years after amendment application, it is difficult to predict whether such changes may emerge in the longer term. Experiments involving repeated relatively low amounts of inorganic N addition over time—somewhat analogous to the gradual mineralization of amendment organic N to plant‐available forms, if not a perfect proxy—have found that losses of plant species diversity not detected in the short term may become apparent in the longer term (e.g., after 8 years; Clark & Tilman, 2008). Similar patterns have been observed in response to N deposition (e.g., Dise et al., 2011; Stevens et al., 2010). Unfortunately, few organic amendment addition studies have reported both short‐ and long‐term observations of plant species diversity. The one study we found with repeated measurements of this nature (Gazol et al., 2016) did find increasing species loss over time following biosolids addition, which trait analyses suggested was due to competitive exclusion. Additionally, Blumenthal et al. (2017), although not measuring plant species diversity, reported increasing relative biomass of the invasive *Bromus tectorum* when repeatedly measured over time in plots amended with composted manure; *B. tectorum* has elsewhere been linked with plant diversity loss (Duncan et al., 2004). Nevertheless, the longest‐term plant diversity measurements we found for low‐N amendments (0.70%–1.85% N), made after 4–23 years, showed a mixture of results (Bastida et al., 2013; González Polo, Kowaljow, Castán, Sauzet, & Mazzarino, 2015; Walter, Martínez, & Cuevas, 2006), suggesting that there may be some threshold amendment N content below which plant species diversity decreases would not be expected, even in the long term. However, use of such low‐N amendments may also result in reduced ANPP benefits. More long‐term observations of plant species diversity and ANPP in low‐N‐amendment‐addition plots are needed.

An additional caveat for interpreting the plant species diversity results is that attributes of plant communities important to biodiversity conservation and other goals on rangelands—for example, cover of exotic relative to native species—may be changing even when plant species diversity is not, with potential consequences for ecosystem stability and function (Avolio et al., 2014). We extracted data on the percent cover of grasses vs. forbs, exotics vs. natives, and annuals vs. perennials, but most of the observations could not be used because they lacked variance information. In the observations that could be analyzed, we observed a trend toward greater cover of annuals relative to perennials, which would be a negative conservation outcome in at least some rangeland systems (Newman, Krzic, & Wallace, 2014; Stromberg, Corbin, & D'Antonio, 2007).

Furthermore, grasslands in Mediterranean systems in particular tend to be exceptionally botanically diverse with many endemic and spatially restricted species at high risk of extirpation due to land use change and management (Cowling, Rundel, Lamont, Arroyo, & Arianoutsou, 1996). However, although soil (notably poor soil) and climate are known to be key drivers of community composition, the environmental determinants of plant species distribution and richness are not fully understood at fine scales (Baldwin et al., 2017), making it difficult to predict how amendment addition may affect landscape‐level diversity. In addition, because these systems exhibit nonequilibrium characteristics (Spiegal, Huntsinger, Hopkinson, & Bartolome, 2016), system changes, including species loss, are often difficult or impossible to undo. Taken together, these ecological characteristics suggest a precautionary approach to organic amendment application might be appropriate for diverse native grasslands.

### 4.6 Runoff quantity and quality

A beneficial outcome from several perspectives was the steep reduction in runoff observed, which would benefit water storage in rangeland soils, in turn supporting resilient forage productivity, as well as benefitting surface water quality. However, particularly in areas where infiltration has not been degraded by past management, it is possible that substantial increases in infiltration and decreases in runoff over large areas could alter stream flows, with potential consequences for aquatic wildlife and humans who depend on those water supplies. It is therefore important to evaluate predicted changes in runoff in the context of the overall hydrology and management goals of the application site.

The steep reduction in runoff brought about by amendments may mitigate the increases in runoff concentrations of nitrate and P that they also tend to cause, such that nutrient exports from amended vs. unamended areas might be comparable. Unfortunately, we could not quantify nutrient export effect sizes, because the majority of studies provided runoff quantity and nutrient concentration information separately (i.e., with no variance information for export). Nutrient concentrations from soil leachates were also outcomes of interest, as potential nutrient transfers to groundwater are another possible harm that could result from amendment addition. However, as we found no studies using field lysimeters or other appropriate methods to measure this outcome, it remains an important data gap.

Organic amendments can reduce runoff in at least two major ways: the presence of the amendment itself on the soil surface can increase infiltration and water holding capacity, and the amendment can boost vegetation growth which slows surface water movement and increases infiltration. Within the particularly short time frame of many runoff studies (median application to measurement time = 6 months), increasing the amount of amendment applied provided a greater relative runoff reduction benefit for low‐N amendments compared to high‐N amendments (Supporting Information Figure S1d). This suggests that more amendment might provide a greater relative boost for the former runoff reduction mechanism (effect of amendment itself on infiltration and water holding capacity) than for the latter mechanism (stimulation of vegetation growth by the amendment), although these patterns might differ over the longer term. Interestingly, soil organic C effect size showed a parallel pattern for the interaction between amount of amendment applied and amendment N concentration (Supporting Information Figure S1c), as might be expected if amendment decomposition patterns were driving total soil organic C measurements at the time when most measurements were taken, and lower N amendments were decomposing more slowly (Averill & Waring, 2018; Melillo et al., 1982; Zhang et al., 2008). Nevertheless, the association of higher amendment N with a greater amount of runoff reduction overall (Table 4A) suggests that amendments with greater ability to stimulate vegetation growth might ultimately provide more runoff reduction.

### 4.7 Applications

With growing interest in organic amendment application to rangelands—especially as a mitigation and adaptation strategy to address global change impacts such as dryland expansion and degradation—understanding ecosystem‐wide outcomes of these applications is increasingly important. We found that organic amendment application, at least as it has been studied to date, results in mixtures of benefits and harms, with low‐N amendments helping to maximize benefits and minimize harms according to the specific definitions and framework we used. Climate zone, the only site descriptor included in our explanatory models, had some significant interactions with other predictors, suggesting that the same amendment application might result in somewhat different patterns of benefits at dryland as compared to Mediterranean sites and that different results might be found in climate zones other than those we analyzed.

At the same time as outcomes of amendment application can be context dependent, so too can the values that landowners assign to different benefits and harms. Given the diversity of rangeland management goals (Roche et al., 2015), we recommend a careful matching of amendment application strategy to rangeland site context. For example, in a stream‐dissected rangeland with high native plant diversity, managers who value water quality and biodiversity may decide to forego amendment application. In a rangeland area where heavy grazing has reduced plant cover and increased runoff, but which still has potential to support native species, a low‐N amendment might be preferred. On a retired agricultural field that has been converted to livestock pasture use, managers may apply higher amounts of whatever amendments are available and affordable so that benefits such as greater ANPP can be realized quickly. Tailoring amendment application strategies to initial site conditions and future site goals would also be important for restoration or rehabilitation projects—for example, converting former mines into grasslands—where there is often considerable potential for benefits (Ohsowski, Klironomos, Dunfield, & Hart, 2012), but also some risk of harms, if, for example, high‐fertility conditions coincide with low plant cover.

Given the multifunctionality of rangeland landscapes, many rangeland managers are familiar with the process of weighing potential benefits and harms of new management practices across multiple economic and ecological outcomes. Quantitative analyses synthesizing a practice's effect on multiple outcomes—such as the one we have presented here—can provide important assistance with this process. These analyses can also identify critical data gaps, such as the urgent need for more long‐term studies and more comprehensive assessments of climate change mitigation potential; indeed, confidence in predicting long‐term outcomes of rangeland amendment application will remain limited until such data are available. For broader planning efforts, additional studies comparing the potential of this practice vs. others to meet identified goals will provide important context for our findings. In addition, more studies comparing the benefits and harms of applying amendments to rangelands vs. croplands, pastures, restoration sites, or urban lands would be valuable, especially since some potential harms, such as plant diversity impacts, may be less of a concern on these other land types. Ultimately, leveraging this information to develop site‐appropriate strategies will enable potential opportunities from rangeland management practices such as organic amendment addition to be realized while minimizing risks.

## References

[gcb14535-bib-0002] Aguilar, R. , & Loftin, S. R. (1992). Sewage sludge application in semiarid grasslands: Effects on runoff and surface water quality In Klett C. T. O. (Ed.), Proceedings of the 36th annual New Mexico water conference: Agencies and science working for the future. WRRI Report No. 265 (pp. 101–111). Las Cruces, NM: New Mexico Water Resources Research Institute.

[gcb14535-bib-0003] Albaladejo, J. , Castillo, V. , & Díaz, E. (2000). Soil loss and runoff on semiarid land as amended with urban solid refuse. Land Degradation and Development, 11(4), 363–373. 10.1002/1099-145X(200007/08)11:4<363:AID-LDR399>3.0.CO;2-R

[gcb14535-bib-0004] Anderson, E. W. (1993). Prescription grazing to enhance rangeland watersheds. Rangelands, 15(1), 31–35.

[gcb14535-bib-0005] Averill, C. , & Waring, B. (2018). Nitrogen limitation of decomposition and decay: How can it occur? Global Change Biology, 24(4), 1417–1427. 10.1111/gcb.13980 29121419

[gcb14535-bib-0006] Avolio, M. L. , Koerner, S. E. , La Pierre, K. J. , Wilcox, K. R. , Wilson, G. W. T. , Smith, M. D. , & Collins, S. L. (2014). Changes in plant community composition, not diversity, during a decade of nitrogen and phosphorus additions drive above‐ground productivity in a tallgrass prairie. Journal of Ecology, 102(6), 1649–1660. 10.1111/1365-2745.12312

[gcb14535-bib-0007] Baldwin, B. G. , Thornhill, A. H. , Freyman, W. A. , Ackerly, D. D. , Kling, M. M. , Morueta‐Holme, N. , & Mishler, B. D. (2017). Species richness and endemism in the native flora of California. American Journal of Botany, 104(3), 487–501. 10.3732/ajb.1600326 28341628

[gcb14535-bib-0008] Bartley, R. , Roth, C. H. , Ludwig, J. , McJannet, D. , Liedloff, A. , Corfield, J. , … Abbott, B. (2006). Runoff and erosion from Australia’s tropical semi‐arid rangelands: Influence of ground cover for differing space and time scales. Hydrological Processes, 20, 3317–3333. 10.1002/hyp.6334

[gcb14535-bib-0009] Bastida, F. , Hernández, T. , Albaladejo, J. , & García, C. (2013). Phylogenetic and functional changes in the microbial community of long‐term restored soils under semiarid climate. Soil Biology and Biochemistry, 65, 12–21. 10.1016/j.soilbio.2013.04.022

[gcb14535-bib-0010] Bastida, F. , Kandeler, E. , Moreno, J. L. , Ros, M. , García, C. , & Hernández, T. (2008). Application of fresh and composted organic wastes modifies structure, size and activity of soil microbial community under semiarid climate. Applied Soil Ecology, 40(2), 318–329. 10.1016/j.apsoil.2008.05.007

[gcb14535-bib-0011] Bastida, F. , Moreno, J. L. , García, C. , & Hernández, T. (2007). Addition of urban waste to semiarid degraded soil: Long‐term effect. Pedosphere, 17(5), 557–567. 10.1016/S1002-0160(07)60066-6

[gcb14535-bib-0012] Bastida, F. , Selevsek, N. , Torres, I. F. , Hernández, T. , & García, C. (2015). Soil restoration with organic amendments: Linking cellular functionality and ecosystem processes. Scientific Reports, 5, 1–12. 10.1038/srep15550 PMC4621494 26503516

[gcb14535-bib-0013] Bedunah, D. J. , & Angerer, J. P. (2012). Rangeland degradation, poverty, and conflict: How can rangeland scientists contribute to effective responses and solutions? Rangeland Ecology and Management, 65(6), 606–612. 10.2111/REM-D-11-00155.1

[gcb14535-bib-0014] Blumenthal, D. M. , Lecain, D. R. , & Augustine, D. J. (2017). Composted manure application promotes long‐term invasion of semi‐arid rangeland by *Bromus tectorum* . Ecosphere, 8(10), e01960 10.1002/ecs2.1960

[gcb14535-bib-0015] Booker, K. , Huntsinger, L. , Bartolome, J. W. , Sayre, N. F. , & Stewart, W. (2013). What can ecological science tell us about opportunities for carbon sequestration on arid rangelands in the United States? Global Environmental Change, 23(1), 240–251. 10.1016/j.gloenvcha.2012.10.001

[gcb14535-bib-0016] Borer, E. T. , Seabloom, E. W. , Gruner, D. S. , Harpole, W. S. , Hillebrand, H. , Lind, E. M. , … Yang, L. H. (2014). Herbivores and nutrients control grassland plant diversity via light limitation. Nature, 508(7497), 517–520. 10.1038/nature13144 24670649

[gcb14535-bib-0017] Brown, J. R. , & Herrick, J. E. (2016). Making soil health a part of rangeland management. Journal of Soil and Water Conservation, 71(3), 55A–60A. 10.2489/jswc.71.3.55A

[gcb14535-bib-0018] Cabrera, V. E. , Stavast, L. J. , Baker, T. T. , Wood, M. K. , Cram, D. S. , Flynn, R. P. , & Ulery, A. L. (2009). Soil and runoff response to dairy manure application on New Mexico rangeland. Agriculture, Ecosystems and Environment, 131(3–4), 255–262. 10.1016/j.agee.2009.01.022

[gcb14535-bib-0019] Cáceres, D. M. , Tapella, E. , Quétier, F. , & Díaz, S. (2015). The social value of biodiversity and ecosystem services from the perspectives of different social actors. Ecology and Society, 20(1), 62 10.5751/ES-07297-200162

[gcb14535-bib-0020] Calcagno, V. (2013). glmulti: Model selection and multimodel inference made easy. R package version 1.0.7. Retrieved from https://CRAN.R-project.org/package=glmulti

[gcb14535-bib-0021] Cameron, D. R. , Marty, J. , & Holland, R. F. (2014). Whither the rangeland?: Protection and conversion in California’s rangeland ecosystems. PLoS ONE, 9(8), e103468 10.1371/journal.pone.0103468 25141171 PMC4139198

[gcb14535-bib-0022] Cameron, D. R. , Marvin, D. C. , Remucal, J. M. , & Passero, M. C. (2017). Ecosystem management and land conservation can substantially contribute to California’s climate mitigation goals. Proceedings of the National Academy of Sciences of the United States of America, 114(48), 12833–12838. 10.1073/pnas.1707811114 29133408 PMC5715745

[gcb14535-bib-0023] Caravaca, F. , Figueroa, D. , Alguacil, M. M. , & Roldán, A. (2003). Application of composted urban residue enhanced the performance of afforested shrub species in a degraded semiarid land. Bioresource Technology, 90(1), 65–70. 10.1016/S0960-8524(03)00087-7 12835059

[gcb14535-bib-0024] Caravaca, F. , Figueroa, D. , Azcón‐Aguilar, C. , Barea, J. M. , & Roldán, A. (2003). Medium‐term effects of mycorrhizal inoculation and composted municipal waste addition on the establishment of two Mediterranean shrub species under semiarid field conditions. Agriculture, Ecosystems and Environment, 97(1–3), 95–105. 10.1016/S0167-8809(03)00126-9

[gcb14535-bib-0025] Caravaca, F. , Garcia, C. , Hernández, M. T. , & Roldán, A. (2002). Aggregate stability changes after organic amendment and mycorrhizal inoculation in the afforestation of a semiarid site with *Pinus halepensis* . Applied Soil Ecology, 19(3), 199–208. 10.1016/S0929-1393(01)00189-5

[gcb14535-bib-0026] Clark, C. M. , & Tilman, D. (2008). Loss of plant species after chronic low‐level nitrogen deposition to prairie grasslands. Nature, 451(7179), 712–715. 10.1038/nature06503 18256670

[gcb14535-bib-0027] Conley, D. J. , Paerl, H. W. , Howarth, R. W. , Boesch, D. F. , Seitzinger, S. P. , Havens, K. E. , … Likens, G. E. (2009). Controlling eutrophication: Nitrogen and phosphorus. Science, 323, 1014–1015. 10.1126/science.1167755 19229022

[gcb14535-bib-0028] R Core Team (2018). R: A language and environment for statistical computing. Vienna, Austria: R Foundation for Statistical Computing Retrieved from https://www.r-project.org/

[gcb14535-bib-0029] Cowling, R. M. , Rundel, P. W. , Lamont, B. B. , Arroyo, M. K. , & Arianoutsou, M. (1996). Plant diversity in mediterranean‐climate regions. Trends in Ecology and Evolution, 11(9), 362–366. 10.1016/0169-5347(96)10044-6 21237880

[gcb14535-bib-0030] Crohn, D. M. , Chaganti, V. N. , & Reddy, N. (2013). Composts as post‐fire erosion treatments and their effect on runoff water quality. Transactions of the ASABE, 56(2), 423–435. 10.13031/2013.42692

[gcb14535-bib-0031] Cross, A. , & Sohi, S. P. (2011). The priming potential of biochar products in relation to labile carbon contents and soil organic matter status. Soil Biology and Biochemistry, 43(10), 2127–2134. 10.1016/j.soilbio.2011.06.016

[gcb14535-bib-0032] de Vries, W. , Römkens, P. F. A. M. , & Schütze, G. . (2007). Critical soil concentrations of cadmium, lead, and mercury in view of health effects on humans and animals In Ware G. W. & Whitacre D. M. (Eds.), Reviews of environmental contamination and toxicology (Vol. 191, pp. 91–130). New York, NY: Springer. 10.1007/978-0-387-69163-3_4 17708073

[gcb14535-bib-0033] DeLonge, M. S. , Ryals, R. , & Silver, W. L. (2013). A lifecycle model to evaluate carbon sequestration potential and greenhouse gas dynamics of managed grasslands. Ecosystems, 16(6), 962–979. 10.1007/s10021-013-9660-5

[gcb14535-bib-0034] Dennis, G. L. , & Fresquez, P. R. (1989). The soil microbial community in a sewage‐sludge‐amended semi‐arid grassland. Biology and Fertility of Soils, 7(4), 310–317. 10.1007/BF00257825

[gcb14535-bib-0035] Derner, J. D. , Smart, A. J. , Toombs, T. P. , Larsen, D. , McCulley, R. L. , Goodwin, J. , … Roche, L. M. (2018). Soil health as a transformational change agent for U.S. grazing lands management. Rangeland Ecology and Management, 71(4), 403–408. 10.1016/j.rama.2018.03.007

[gcb14535-bib-0036] Diacono, M. , & Montemurro, F. (2010). Long‐term effects of organic amendments on soil fertility. A review. Agronomy for Sustainable Development, 30, 401–422. 10.1051/agro/2009040

[gcb14535-bib-0037] Díaz‐Raviña, M. , Martín, A. , Barreiro, A. , Lombao, A. , Iglesias, L. , Díaz‐Fierros, F. , & Carballas, T. (2012). Mulching and seeding treatments for post‐fire soil stabilisation in NW Spain: Short‐term effects and effectiveness. Geoderma, 191, 31–39. 10.1016/j.geoderma.2012.01.003

[gcb14535-bib-0038] Dise, N. B. , Ashmore, M. R. , Belyazid, S. , Bobbink, R. , DeVries, W. , Erisman, J. W. , … van den Berg, L. (2011). Nitrogen as a threat to European terrestrial biodiversity In Sutton M. A., Howard C. M., Erisman J. W., Billen G., Bleeker A., Grennfelt P., … Grizzetti B. (Eds.), The European nitrogen assessment (pp. 463–494). Cambridge, UK: Cambridge University Press.

[gcb14535-bib-0039] Duncan, C. A. , Jachetta, J. J. , Brown, M. L. , Carrithers, V. F. , Clark, J. K. , DiTomaso, J. M. , … Rice, P. M. (2004). Assessing the economic, environmental, and societal losses from invasive plants on rangeland and wildlands. Weed Technology, 18(sp1), 1411–1416. 10.1614/0890-037X(2004)018

[gcb14535-bib-0040] Duniway, M. C. , Herrick, J. E. , & Monger, H. C. (2010). Spatial and temporal variability of plant‐available water in calcium carbonate‐cemented soils and consequences for arid ecosystem resilience. Oecologia, 163(1), 215–226. 10.1007/s00442-009-1530-7 20020157

[gcb14535-bib-0041] Everaert, G. , Deschutter, Y. , De Troch, M. , Janssen, C. R. , & De Schamphelaere, K. (2018). Multimodel inference to quantify the relative importance of abiotic factors in the population dynamics of marine zooplankton. Journal of Marine Systems, 181, 91–98. 10.1016/j.jmarsys.2018.02.009

[gcb14535-bib-0042] Fenn, M. E. , Allen, E. B. , Weiss, S. B. , Jovan, S. , Geiser, L. H. , Tonnesen, G. S. , … Bytnerowicz, A. (2010). Nitrogen critical loads and management alternatives for N‐impacted ecosystems in California. Journal of Environmental Management, 91(12), 2404–2423. 10.1016/j.jenvman.2010.07.034 20705383

[gcb14535-bib-0043] Follett, R. F. , & Reed, D. A. (2010). Soil carbon sequestration in grazing lands: Societal benefits and policy implications. Rangeland Ecology and Management, 63(1), 4–15. 10.2111/08-225.1

[gcb14535-bib-0044] Fresquez, P. R. , Francis, R. , & Dennis, G. L. (1990). Soil and vegetation responses to sewage sludge on a degraded semiarid broom snakeweed / blue grama plant community. Journal of Range Management, 43(4), 325–331. 10.2307/3898926

[gcb14535-bib-0045] Friedel, M. H. (1991). Range condition assessment and the concept of thresholds: A viewpoint. Journal of Range Management, 44(5), 422–426. 10.2307/4002737

[gcb14535-bib-0046] Fuentes, D. , Valdecantos, A. , Llovet, J. , Cortina, J. , & Vallejo, V. R. (2010). Fine‐tuning of sewage sludge application to promote the establishment of *Pinus halepensis* seedlings. Ecological Engineering, 36(10), 1213–1221. 10.1016/j.ecoleng.2010.04.012

[gcb14535-bib-0047] Fynn, A. J. , Alvarez, P. , Brown, J. R. , George, M. R. , Kustin, C. , Laca, E. A. , … Wong, C. P. (2009). Soil carbon sequestration in U.S. rangelands: Issues paper for protocol development. New York. Retrieved from http://www.fao.org/fileadmin/templates/agphome/scpi/cgwg/Soil_Carbon_US_Rangelands_IP.pdf

[gcb14535-bib-0048] Garnett, T. , Godde, C. , Muller, A. , Röös, E. , Smith, P. , de Boer, I. , … van Zanten, H. (2017). Grazed and confused? Ruminating on cattle, grazing systems, methane, nitrous oxide, the soil carbon sequestration question – And what it all means for greenhouse gas emissions. Oxford. Retrieved fromhttps://www.fcrn.org.uk/sites/default/files/project-files/fcrn_gnc_report.pdf

[gcb14535-bib-0049] Gazol, A. , Uria‐Diez, J. , Elustondo, D. , Garrigó, J. , & Ibáñez, R. (2016). Fertilization triggers 11 years of changes in community assembly in Mediterranean grassland. Journal of Vegetation Science, 27(4), 728–738. 10.1111/jvs.12409

[gcb14535-bib-0050] Gea‐Izquierdo, G. , Gennet, S. , & Bartolome, J. W. (2007). Assessing plant‐nutrient relationships in highly invaded Californian grasslands using non‐normal probability distributions. Applied Vegetation Science, 10(3), 343–350. 10.1111/j.1654-109X.2007.tb00433.x

[gcb14535-bib-0051] González, A. L. , Kominoski, J. S. , Danger, M. , Ishida, S. , Iwai, N. , & Rubach, A. (2010). Can ecological stoichiometry help explain patterns of biological invasions? Oikos, 119(5), 779–790. 10.1111/j.1600-0706.2009.18549.x

[gcb14535-bib-0052] González Polo, M. , Kowaljow, E. , Castán, E. , Sauzet, O. , & Mazzarino, M. J. (2015). Persistent effect of organic matter pulse on a sandy soil of semiarid Patagonia. Biology and Fertility of Soils, 51(2), 241–249. 10.1007/s00374-014-0961-4

[gcb14535-bib-0053] Goss, M. J. , Tubeileh, A. , & Goorahoo, D. (2013). A review of the use of organic amendments and the risk to human health In Sparks D. L. (Ed.), Advances in agronomy (Vol. 120, pp. 275–379). Amsterdam, The Netherlands: Elsevier.

[gcb14535-bib-0054] Gravuer, K. , & Gunasekara, A. (2016). Compost application rates for California croplands and rangelands for a CDFA Healthy Soils Incentives Program. Sacramento, California. Retrieved from https://www.cdfa.ca.gov/oefi/efasap/docs/CompostApplicationRate_WhitePaper.pdf

[gcb14535-bib-0055] Haden, V. R. , De Gryze, S. , & Nelson, N. (2014). American Carbon Registry methodology for compost additions to grazed grasslands. Arlington, VA. Retrieved from https://americancarbonregistry.org/carbon-accounting/standards-methodologies/methodology-for-greenhouse-gas-emission-reductions-from-compost-additions-to-grazed-grasslands

[gcb14535-bib-0056] Haferkamp, M. R. , Grings, E. E. , Heitschmidt, R. K. , MacNeil, M. D. , & Karl, M. G. (2001). Suppression of annual bromes impacts rangeland: Animal responses. Journal of Range Management, 54(6), 663–668. 10.2307/4003668

[gcb14535-bib-0057] Hanke, W. , Gröngröft, A. , Jürgens, N. , & Schmiedel, U. (2011). Rehabilitation of arid rangelands: Intensifying water pulses from low‐intensity winter rainfall. Journal of Arid Environments, 75(2), 185–193. 10.1016/j.jaridenv.2010.09.002

[gcb14535-bib-0058] Hargreaves, J. C. , Adl, M. S. , & Warman, P. R. (2008). A review of the use of composted municipal solid waste in agriculture. Agriculture, Ecosystems and Environment, 123(1–3), 1–14. 10.1016/j.agee.2007.07.004

[gcb14535-bib-0059] Harpole, W. S. , Sullivan, L. L. , Lind, E. M. , Firn, J. , Adler, P. B. , Borer, E. T. , … Wragg, P. D. (2016). Addition of multiple limiting resources reduces grassland diversity. Nature, 537, 93–96. 10.1038/nature19324 27556951

[gcb14535-bib-0060] Havstad, K. M. , Peters, D. P. C. , Skaggs, R. , Brown, J. , Bestelmeyer, B. , Fredrickson, E. , … Wright, J. (2007). Ecological services to and from rangelands of the United States. Ecological Economics, 64(2), 261–268. 10.1016/j.ecolecon.2007.08.005

[gcb14535-bib-0061] Hedges, L. V. , Gurevitch, J. , & Curtis, P. S. (1999). The meta‐analysis of response ratios in experimental ecology. Ecology, 80(4), 1150–1156. 10.2307/177062

[gcb14535-bib-0062] Herrick, J. E. , & Wander, M. M. (1998). Relationships between soil organic carbon and soil quality in cropped and rangeland soils: The importance of distribution, composition, and soil biological activity In Lal R., Kimble J. M., Follett R. F., & Stewart B. A. (Eds.), Soil processes and the carbon cycle (pp. 405–425). Boca Raton, FL: CRC Press.

[gcb14535-bib-0063] Hill, J. (2005). Recycling biosolids to pasture‐based animal production systems in Australia: A review of evidence on the control of potentially toxic metals and persistent organic compounds recycled to agricultural land. Australian Journal of Agricultural Research, 56, 753–773. 10.1071/AR04264

[gcb14535-bib-0064] Hoekstra, J. M. , Boucher, T. M. , Ricketts, T. H. , & Roberts, C. (2005). Confronting a biome crisis: Global disparities of habitat loss and protection. Ecology Letters, 8(1), 23–29. 10.1111/j.1461-0248.2004.00686.x

[gcb14535-bib-0065] Huang, J. , Li, Y. , Fu, C. , Chen, F. , Fu, Q. , Dai, A. , … Wang, G. (2017). Dryland climate change: Recent progress and challenges. Reviews of Geophysics, 55, 719–778. 10.1053/j.jvca.2010.06.032

[gcb14535-bib-0066] Huntsinger, L. , Bartolome, J. W. , & D’Antonio, C. M. (2007). Grazing management on California’s Mediterranean grasslands In Stromberg M. R., Corbin J. D., & D’Antonio C. M. (Eds.), California grasslands: Ecology and management (pp. 233–253). Berkeley, CA: University of California Press.

[gcb14535-bib-0067] Intergovernmental Panel on Climate Change (2014). Climate change 2014: Synthesis report. Contribution of Working Groups I, II and III to the Fifth Assessment Report of the Intergovernmental Panel on Climate Change [Core Writing Team, R.K. Pachauri and L.A. Meyer (eds.)]. Geneva, Switzerland. Retrieved from https://www.ipcc.ch/report/ar5/syr/

[gcb14535-bib-0068] Ippolito, J. A. , Barbarick, K. A. , Paschke, M. W. , & Brobst, R. B. (2010). Infrequent composted biosolids applications affect semi‐arid grassland soils and vegetation. Journal of Environmental Management, 91(5), 1123–1130. 10.1016/j.jenvman.2010.01.004 20097468

[gcb14535-bib-0069] Isbell, F. , Reich, P. B. , Tilman, D. , Hobbie, S. E. , Polasky, S. , & Binder, S. (2013). Nutrient enrichment, biodiversity loss, and consequent declines in ecosystem productivity. Proceedings of the National Academy of Sciences of the United States of America, 110(29), 11911–11916. 10.1073/pnas.1310880110 23818582 PMC3718098

[gcb14535-bib-0070] Isbell, F. , Craven, D. , Connolly, J. , Loreau, M. , Schmid, B. , Beierkuhnlein, C. , … Eisenhauer, N. (2015). Biodiversity increases the resistance of ecosystem productivity to climate extremes. Nature, 526(7574), 574–577. 10.1038/nature15374 26466564

[gcb14535-bib-0071] Jurado‐Guerra, P. , Luna‐Luna, M. , Flores‐Ancira, E. , & Saucedo‐Teran, R. (2013). Residual effects of biosolids application on forage production of semiarid grassland in Jalisco, Mexico. Applied and Environmental Soil Science, 2013, Article ID 835960. 10.1155/2013/835960

[gcb14535-bib-0072] Khaleel, R. , Reddy, K. R. , & Overcash, M. R. (1981). Changes in soil physical properties due to organic waste applications: A review. Journal of Environmental Quality, 10(2), 133–141. 10.2134/jeq1981.00472425001000020002x

[gcb14535-bib-0073] Kowaljow, E. , Mazzarino, M. J. , Satti, P. , & Jiménez‐Rodríguez, C. (2010). Organic and inorganic fertilizer effects on a degraded Patagonian rangeland. Plant and Soil, 332, 135–145. 10.1007/s11104-009-0279-4

[gcb14535-bib-0074] Kuzyakov, Y. (2010). Priming effects: Interactions between living and dead organic matter. Soil Biology and Biochemistry, 42(9), 1363–1371. 10.1016/j.soilbio.2010.04.003

[gcb14535-bib-0075] Lai, L. , Wang, J. , Tian, Y. , Zhao, X. , Jiang, L. , Chen, X. , … Zheng, Y. (2013). Organic matter and water addition enhance soil respiration in an arid region. PLoS ONE, 8(10), e77659 10.1371/journal.pone.0077659 24204907 PMC3799695

[gcb14535-bib-0076] Lal, R. (2018). Digging deeper: A holistic perspective of factors affecting soil organic carbon sequestration in agroecosystems. Global Change Biology, 24, 3285–3301. 10.1111/gcb.14054 29341449

[gcb14535-bib-0077] Larney, F. J. , & Angers, D. A. (2012). The role of organic amendments in soil reclamation: A review. Canadian Journal of Soil Science, 92(1), 19–38. 10.4141/cjss2010-064

[gcb14535-bib-0078] Louro, A. , Cárdenas, L. M. , García, M. I. , & Báez, D. (2016). Greenhouse gas fluxes from a grazed grassland soil after slurry injections and mineral fertilizer applications under the Atlantic climatic conditions of NW Spain. Science of the Total Environment, 573, 258–269. 10.1016/j.scitotenv.2016.08.092 27567389

[gcb14535-bib-0079] Ludwig, J. , Wilcox, B. P. , Breshears, D. D. , Tongway, D. J. , & Imeson, A. C. (2005). Vegetation patches and runoff‐erosion as interacting ecohydrological processes in semiarid landscapes. Ecology, 86(2), 288–297. 10.1890/03-0569

[gcb14535-bib-0080] Manzetti, S. , & van der Spoel, D. (2015). Impact of sludge deposition on biodiversity. Ecotoxicology, 24(9), 1799–1814. 10.1007/s10646-015-1530-9 26318179

[gcb14535-bib-0081] Martínez, F. , Cuevas, G. , Calvo, R. , & Walter, I. (2003). Biowaste effects on soil and native plants in a semiarid ecosystem. Journal of Environmental Quality, 32(2), 472–479. 10.2134/jeq2003.4720 12708670

[gcb14535-bib-0082] Melillo, J. M. , Aber, J. D. , & Muratore, J. F. (1982). Nitrogen and lignin control of hardwood leaf litter decomposition dynamics. Ecology, 63(3), 621–626. 10.2307/1936780

[gcb14535-bib-0001] National Academies of Sciences, Engineering, and Medicine (2016). Nutrient requirements of beef cattle, eighth revised edition. Washington, DC: The National Academies Press. 38386771

[gcb14535-bib-0083] National Research Council (2002). Biosolids applied to land: Advancing standards and practices. Washington, DC: National Academies Press.

[gcb14535-bib-0084] Newman, R. F. , Krzic, M. , & Wallace, B. M. (2014). Differing effects of biosolids on native plants in grasslands of southern British Columbia. Journal of Environmental Quality, 43(5), 1672 10.2134/jeq2014.01.0013 25603253

[gcb14535-bib-0085] Nichols, K. L. , Del Grosso, S. J. , Derner, J. D. , Follett, R. F. , Archibeque, S. L. , Stewart, C. E. , & Paustian, K. H. (2016). Nitrous oxide and methane fluxes from cattle excrement on C3 pasture and C4‐dominated shortgrass steppe. Agriculture, Ecosystems and Environment, 225, 104–115. 10.1016/j.agee.2016.03.026

[gcb14535-bib-0086] Nicolás, C. , Kennedy, J. N. , Hernández, T. , García, C. , & Six, J. (2014). Soil aggregation in a semiarid soil amended with composted and non‐composted sewage sludge‐A field experiment. Geoderma, 219–220, 24–31. 10.1016/j.geoderma.2013.12.017

[gcb14535-bib-0087] Ochoa‐Hueso, R. , Allen, E. B. , Branquinho, C. , Cruz, C. , Dias, T. , Fenn, M. E. , … Stock, W. D. (2011). Nitrogen deposition effects on Mediterranean‐type ecosystems: An ecological assessment. Environmental Pollution, 159(10), 2265–2279. 10.1016/j.envpol.2010.12.019 21277663

[gcb14535-bib-0088] Ohsowski, B. M. , Klironomos, J. N. , Dunfield, K. E. , & Hart, M. M. (2012). The potential of soil amendments for restoring severely disturbed grasslands. Applied Soil Ecology, 60, 77–83. 10.1016/j.apsoil.2012.02.006

[gcb14535-bib-0089] Ojeda, G. , Alcañiz, J. M. , & Le Bissonnais, Y. (2008). Differences in aggregate stability due to various sewage sludge treatments on a Mediterranean calcareous soil. Agriculture, Ecosystems and Environment, 125(1–4), 48–56. 10.1016/j.agee.2007.11.005

[gcb14535-bib-0090] Paustian, K. , Lehmann, J. , Ogle, S. , Reay, D. , Robertson, G. P. , & Smith, P. (2016). Climate‐smart soils. Nature, 532(7597), 49–57. 10.1038/nature17174 27078564

[gcb14535-bib-0091] Peel, M. C. , Finlayson, B. L. , & McMahon, T. A. (2007). Updated world map of the Köppen‐Geiger climate classification. Hydrology and Earth System Sciences, 11, 1633–1644. 10.5194/hess-11-1633-2007

[gcb14535-bib-0092] Pimentel, D. (2006). Soil erosion: A food and environmental threat. Environment, Development and Sustainability, 8(1), 119–137. 10.1007/s10668-005-1262-8

[gcb14535-bib-0093] Quan, C. , Han, S. , Utescher, T. , Zhang, C. , & Liu, Y. S. C. (2013). Validation of temperature‐precipitation based aridity index: Paleoclimatic implications. Palaeogeography, Palaeoclimatology, Palaeoecology, 386, 86–95. 10.1016/j.palaeo.2013.05.008

[gcb14535-bib-0094] Roche, L. M. , Schohr, T. K. , Derner, J. D. , Lubell, M. N. , Cutts, B. B. , Kachergis, E. , … Tate, K. W. (2015). Sustaining working rangelands: Insights from rancher decision making. Rangeland Ecology and Management, 68(5), 383–389. 10.1016/j.rama.2015.07.006

[gcb14535-bib-0095] Rohatgi, A. (2017). WebPlotDigitizer. Retrieved from https://automeris.io/WebPlotDigitizer

[gcb14535-bib-0096] Ros, M. , Garcia, C. , & Hernandez, T. (2001). The use of urban organic wastes in the control of erosion in a semiarid Mediterranean soil. Soil Use and Management, 17(4), 292–293. 10.1111/j.1475-2743.2001.tb00041.x

[gcb14535-bib-0097] Rostagno, C. M. , & Sosebee, R. E. (2001). Biosolids application in the Chihuahuan Desert: Effects on runoff water quality. Journal of Environmental Quality, 30(1), 160–170. 10.2134/jeq2001.301160x 11215648

[gcb14535-bib-0098] Ryals, R. , Hartman, M. D. , Parton, W. J. , DeLonge, M. S. , & Silver, W. L. (2015). Long‐term climate change mitigation potential with organic matter management on grasslands. Ecological Applications, 25(2), 531–545. 10.1890/13-2126.1 26263673

[gcb14535-bib-0099] Ryals, R. , Kaiser, M. , Torn, M. S. , Berhe, A. A. , & Silver, W. L. (2014). Impacts of organic matter amendments on carbon and nitrogen dynamics in grassland soils. Soil Biology and Biochemistry, 68, 52–61. 10.1016/j.soilbio.2013.09.011

[gcb14535-bib-0100] Ryals, R. , & Silver, W. L. (2013). Effects of organic matter amendments on net primary productivity and greenhouse gas emissions in annual grasslands. Ecological Applications, 23(1), 46–59. 10.1890/12-0620.1 23495635

[gcb14535-bib-0101] Sayre, N. F. (2017). The politics of scale: A history of rangeland science. Chicago, IL: University of Chicago Press.

[gcb14535-bib-0102] Sayre, N. F. , McAllister, R. R. J. , Bestelmeyer, B. T. , Moritz, M. , & Turner, M. D. (2013). Earth Stewardship of rangelands: Coping with ecological, economic, and political marginality. Frontiers in Ecology and the Environment, 11(7), 348–354. 10.1890/120333

[gcb14535-bib-0103] Seabloom, E. W. , Borer, E. T. , Buckley, Y. M. , Cleland, E. E. , Davies, K. F. , Firn, J. , … Yang, L. (2015). Plant species origin predicts dominance and response to nutrient enrichment and herbivores in global grasslands. Nature Communications, 6, 7710 10.1038/ncomms8710 PMC4518311 26173623

[gcb14535-bib-0104] Sharpley, A. N. , McDowell, R. W. , & Kleinman, P. J. A. (2001). Phosphorus loss from land to water: Integrating agricultural and environmental management. Plant and Soil, 237(2), 287–307. 10.1023/A:1013335814593

[gcb14535-bib-0105] Sikora, L. J. , & Yakovchenko, V. (1995). Soil organic matter mineralization after compost amendment. Soil Science Society of America Journal, 60(5), 1401–1404. 10.2136/sssaj1996.03615995006000050015x

[gcb14535-bib-0106] Simkin, S. M. , Allen, E. B. , Bowman, W. D. , Clark, C. M. , Belnap, J. , Brooks, M. L. , … Waller, D. M. (2016). Conditional vulnerability of plant diversity to atmospheric nitrogen deposition across the USA. Proceedings of the National Academy of Sciences of the United States of America, 113(15), 4086–4091. 10.5061/dryad.7kn53 27035943 PMC4839424

[gcb14535-bib-0107] Smith, P. (2004). How long before a change in soil organic carbon can be detected? Global Change Biology, 10(11), 1878–1883. 10.1111/j.1365-2486.2004.00854.x

[gcb14535-bib-0108] Spiegal, S. , Huntsinger, L. , Hopkinson, P. , & Bartolome, J. W. (2016). Range ecosystems In Mooney H. A. & Zavaleta E. S. (Eds.), Ecosystems of California (pp. 835–864). Oakland, CA: University of California Press.

[gcb14535-bib-0109] State of California (2016). Healthy soils action plan. Sacramento, CA. Retrieved from https://www.cdfa.ca.gov/oefi/healthysoils/docs/CA-HealthySoilsActionPlan.pdf

[gcb14535-bib-0110] Stavast, L. J. , Baker, T. T. , Ulery, A. L. , Flynn, R. P. , Wood, M. K. , & Cram, D. S. (2005). New Mexico blue grama rangeland response to dairy manure application. Rangeland Ecology & Management, 58(4), 423–429. 10.2111/1551-5028(2005)058[0423:NMBGRR]2.0.CO;2

[gcb14535-bib-0111] Stevens, C. J. , Dise, N. B. , Mountford, J. O. , & Gowing, D. J. (2004). Impact of nitrogen deposition on the species richness of grasslands. Science, 303(5665), 1876–1880. 15031507 10.1126/science.1094678

[gcb14535-bib-0112] Stevens, C. J. , Dupre, C. , Dorland, E. , Gaudnik, C. , Gowing, D. J. G. , Bleeker, A. , … Dise, N. B. (2010). Nitrogen deposition threatens species richness of grasslands across Europe. Environmental Pollution, 158(9), 2940–2945. 10.1016/j.envpol.2010.06.006 20598409

[gcb14535-bib-0113] Stout, W. L. , Weaver, S. R. , Gburek, W. J. , Folmar, G. J. , & Schnabel, R. R. (2000). Water quality implications of dairy slurry applied to cut pastures in the northeast USA. Soil Use and Management, 16(3), 189–193. 10.1111/j.1475-2743.2000.tb00191.x

[gcb14535-bib-0114] Stromberg, M. R. , Corbin, J. D. , & D’Antonio, C. M. (Eds.) (2007). California grasslands: Ecology and management. Berkeley, CA: University of California Press.

[gcb14535-bib-0115] Suding, K. N. , Collins, S. L. , Gough, L. , Clark, C. , Cleland, E. E. , Gross, K. L. , … Pennings, S. (2005). Functional‐ and abundance‐based mechanisms explain diversity loss due to N fertilization. Proceedings of the National Academy of Sciences of the United States of America, 102(12), 4387–4392. 10.1073/pnas.0408648102 15755810 PMC555488

[gcb14535-bib-0116] Sullivan, T. S. , Stromberger, M. E. , & Paschke, M. W. (2006). Parallel shifts in plant and soil microbial communities in response to biosolids in a semi‐arid grassland. Soil Biology and Biochemistry, 38(3), 449–459. 10.1016/j.soilbio.2005.05.018

[gcb14535-bib-0117] Sullivan, T. S. , Stromberger, M. E. , Paschke, M. W. , & Ippolito, J. A. (2006). Long‐term impacts of infrequent biosolids applications on chemical and microbial properties of a semi‐arid rangeland soil. Biology and Fertility of Soils, 42(3), 258–266. 10.1007/s00374-005-0023-z

[gcb14535-bib-0118] Tarrasón, D. , Ojeda, G. , Ortiz, O. , & Alcañiz, J. M. (2010). Effects of different types of sludge on soil microbial properties: A field experiment on degraded Mediterranean soils. Pedosphere, 20(6), 681–691. 10.1016/S1002-0160(10)60058-6

[gcb14535-bib-0119] Tejada, M. , & Gonzalez, J. L. (2008). Influence of two organic amendments on the soil physical properties, soil losses, sediments and runoff water quality. Geoderma, 145, 325–334. 10.1016/j.geoderma.2008.03.020

[gcb14535-bib-0120] Terrer, C. , Vicca, S. , Hungate, B. A. , Phillips, R. P. , & Prentice, I. C. (2016). Mycorrhizal association as a primary control of the CO₂ fertilization effect. Science, 353(6294), 72–74. 10.1126/science.aaf4610 27365447

[gcb14535-bib-0121] Tilman, D. , Reich, P. B. , & Knops, J. M. H. (2006). Biodiversity and ecosystem stability in a decade‐long grassland experiment. Nature, 441(7093), 629–632. 10.1038/nature04742 16738658

[gcb14535-bib-0122] Torres, I. F. , Bastida, F. , Hernández, T. , Albaladejo, J. , & García, C. (2015). Enzyme activity, microbial biomass and community structure in a long‐term restored soil under semi‐arid conditions. Soil Research, 53(5), 553–560. 10.1071/SR14297

[gcb14535-bib-0123] United States Environmental Protection Agency (1993). Standards for the use or disposal of sewage sludge; Final rules. 40 CFR Parts 257, 403 and 503. Federal Register 19 February 1993, 58(32), 9248–9415.

[gcb14535-bib-0124] US Environmental Protection Agency (2005). Ecological soil screening levels for lead: Interim final. Washington, DC. Retrieved from https://www.epa.gov/sites/production/files/2015-09/documents/eco-ssl_lead.pdf

[gcb14535-bib-0125] Viechtbauer, W. (2010). Conducting meta‐analyses in R with the metafor package. Journal of Statistical Software, 36(3), 1–48. 10.18637/jss.v036.i03

[gcb14535-bib-0126] Vucetich, J. A. , Bruskotter, J. T. , & Nelson, M. P. (2015). Evaluating whether nature’s intrinsic value is an axiom of or anathema to conservation. Conservation Biology, 29(2), 321–332. 10.1111/cobi.12464 25704250

[gcb14535-bib-0127] Wallace, B. M. , Krzic, M. , Newman, R. F. , Forge, T. A. , Broersma, K. , & Neilsen, G. (2016). Soil aggregate dynamics and plant community response after biosolids application in a semiarid grassland. Journal of Environmental Quality, 45(5), 1663–1671. 10.2134/jeq2016.01.0030 27695737

[gcb14535-bib-0128] Walter, I. , Martínez, F. , & Cuevas, G. (2006). Plant and soil responses to the application of composted MSW in a degraded, semiarid shrubland in central Spain. Compost Science and Utilization, 14(2), 147–154. 10.1080/1065657X.2006.10702276

[gcb14535-bib-0129] Walton, M. , Herrick, J. E. , Gibbens, R. P. , & Remmenga, M. D. (2001). Persistence of municipal biosolids in a Chihuahuan Desert rangeland 18 years after application. Arid Land Research and Management, 15(3), 223–232. 10.1080/15324980152119784

[gcb14535-bib-0130] Ward, M. H. , deKok, T. M. , Levallois, P. , Brender, J. , Gulis, G. , Nolan, B. T. , & VanDerslice, J. (2005). Workgroup report: Drinking‐water nitrate and health – Recent findings and research needs. Environmental Health Perspectives, 113(11), 1607–1614. 10.1289/ehp.8043 16263519 PMC1310926

[gcb14535-bib-0131] Westoby, M. , Walker, B. , & Noy‐Meir, I. (1989). Opportunistic management for rangelands not at equilibrium. Journal of Range Management, 42(4), 266–274. 10.2307/3899492

[gcb14535-bib-0132] Whitford, W. G. , Aldon, E. F. , Freckman, D. W. , Steinberger, Y. , & Parker, L. W. (1989). Effects of organic amendments on soil biota on a degraded rangeland. Journal of Range Management, 42(1), 56–60. 10.2307/3899659

[gcb14535-bib-0133] Whittingham, M. J. , Krebs, J. R. , Swetnam, R. D. , Thewlis, R. M. , Wilson, J. D. , & Freckleton, R. P. (2009). Habitat associations of British breeding farmland birds. Bird Study, 56(1), 43–52. 10.1080/00063650802648150

[gcb14535-bib-0134] Wilcox, B. P. , Maitre, D. L. , Jobbagy, E. , Wang, L. , & Breshears, D. D. (2017). Ecohydrology: Processes and implications for rangelands In Briske D. D. (Ed.), Rangeland systems: Processes, management and challenges (Springer S, pp. 85–129). Cham, Switzerland: Springer.

[gcb14535-bib-0135] Wolfe, S. A. , & Nickling, W. G. (1993). The protective role of sparse vegetation in wind erosion. Progress in Physical Geography, 17(1), 50–68. 10.1177/030913339301700104

[gcb14535-bib-0136] Xintaras, C. (1992). Impact of lead‐contaminated soil on public health. Atlanta, GA. Retrieved from https://wonder.cdc.gov/wonder/prevguid/p0000015/p0000015.asp

[gcb14535-bib-0137] Yahdjian, L. , Sala, O. E. , & Havstad, K. M. (2015). Rangeland ecosystem services: Shifting focus from supply to reconciling supply and demand. Frontiers in Ecology and the Environment, 13(1), 44–51. 10.1890/140156

[gcb14535-bib-0138] Zhang, D. , Hui, D. , Luo, Y. , & Zhou, G. (2008). Rates of litter decomposition in terrestrial ecosystems: Global patterns and controlling factors. Journal of Plant Ecology, 1(2), 85–93. 10.1093/jpe/rtn002

