# Supplementary material for: Organic amendment additions to rangelands: A meta‐analysis of multiple ecosystem outcomes
Source: Glob Chang Biol. 2019 Jan 2;25(3):1152–70. doi: 10.1111/gcb.14535 (PMC6849820; doi:10.1111/gcb.14535)

Table S1. Characteristics of observations used in explanatory models relating four predictor variables (climate zone, days from application to measurement, amount of amendment applied, and amendment N concentration) to effect sizes measuring the impact of organic amendment addition on each of eight rangeland ecosystem outcomes. As in Table 1, "experiment" refers to a specific set of field plots; publications in which the same treatments were applied at more than one site were considered to contain more than one experiment. "Observation" refers to a unique combination of response variable + experiment + measurement date + amendment type + amount of amendment applied. Number of observations for modeling is often less than number of observations for effect size (Table 1) because, to be included in the modeling data set, an observation had to have data for all four predictor variables, plus soil sample depth for soil outcomes. As noted elsewhere, "dryland" climate refers to Köppen-Geiger climate type B and "Mediterranean" climate refers to Köppen-Geiger climate type Cs. For variables with less than three experiments from Mediterranean climates (i.e., the bottom four rows of the table), climate zone was not used as a predictor variable in modeling.

**Outcome**	**Number of experiments**		**Number of publications**		**Number of observations**	
	Dryland	*Mediterranean*	*Dryland*	*Mediterranean*	*Dryland*	*Mediterranean*
Soil Organic Carbon	16	9	24	11	175	62
Aboveground NPP	13	9	22	10	158	88
Plant Species Diversity	6	3	7	2	69	28
Plant Tissue Nitrogen	6	5	9	7	65	63
Soil Lead (Pb)	6	1	12	1	82	6
Runoff Quantity	10	2	9	1	72	6
Runoff Nitrate	7	2	6	2	38	19
Runoff Phosphorus	7	1	6	1	50	16

Table S1 *(continued)*. Distribution of each predictor variable used in modeling.

**Outcome**	**Days application to measurement quantiles**					**Amount of amendment applied (Mg/ha) quantiles**					**Amendment N concentration (%) quantiles**				
	0	*25*	*50 (median)*	*75*	*100*	*0*	*25*	*50 (median)*	*75*	*100*	*0*	*25*	*50 (median)*	*75*	*100*
Soil Organic Carbon	7	273	556	1461	8400	2.5	10.0	40.0	78.0	260.0	0.70	1.85	3.17	4.20	4.86
Aboveground NPP	24	273	516	901	8400	1.0	9.3	40.0	73.8	260.0	0.70	2.03	2.65	4.15	5.85
Plant Species Diversity	59	424	609	1233	8400	2.5	21.0	45.0	78.0	195.0	0.77	1.87	4.12	4.66	4.86
Plant Tissue Nitrogen	48	273	549.5	1096	4360	1.0	5.0	45.0	90.0	200.0	0.70	2.03	2.46	2.65	5.60
Soil Lead (Pb)	59	365	730.5	1461	6047	21.0	40.0	80.0	95.2	260.0	1.29	1.77	2.03	3.02	4.86
Runoff Quantity	14	95	184	547	1796	7.0	18.0	44.8	90.0	446.0	0.82	2.62	3.36	4.03	6.20
Runoff Nitrate	14	90	366	551	1675	1.5	11.7	34.0	80.0	108.8	0.82	1.77	3.36	4.08	6.20
Runoff Phosphorus	14	95	366	550	1675	1.5	11.7	34.0	79.7	108.8	0.82	2.62	3.51	4.08	6.20

Table S2. Mean values (± SD) of eight outcomes in control (unamended) and treatment (amended) plots. Values are grand means – i.e., means were first calculated for each experiment and the mean of those means is presented so that experiments with more observations did not have disproportionate influence. Standard deviations were calculated for the set of experiment means.

Outcome	Control (unamended) plots mean (± SD)	Treatment (amended) plots mean (± SD)	Units
Soil Organic C	22.4 (± 26.6)	29.2 (± 29.2)	g SOC / kg soil
Aboveground NPP	1717 (± 2165)	3407 (± 4041)	kg / ha*
Plant Species Diversity	14.4 (± 9.7)	13.6 (± 10.8)	number of species per plot**
Plant Tissue N	13.5 (± 5.7)	16.0 (± 6.6)	g N / kg plant tissue
Soil Pb	29.6 (± 23.5)	37.8 (± 20.1)	mg Pb / kg soil***
Runoff Quantity	38.5 (± 31.3)	18.8 (± 25.4)	% of precipitation captured as runoff
Runoff Nitrate	0.159 (± 0.153)	0.889 (± 0.795)	mg NO_3^- / L runoff
Runoff P	0.316 (± 0.325)	2.83 (± 4.59)	mg P / L runoff

* Some studies included in effect size calculation measured percent total vegetation cover rather than vegetation mass; median cover was 52.1% (± 28.8) in control (unamended) plots and 62.5% (± 30.4) in treatment (amended) plots
** Measurements of evenness and combined diversity indices (e.g., Shannon index) were also included in effect size calculation, but are not included here due to their heterogeneous units
*** Some studies included in effect size calculation measured DTPA (diethylenetriaminepentaacetic acid)-extractable Pb rather than total Pb; median DTPA-extractable Pb was 8.1 (± 16.1) mg Pb/kg soil in control (unamended) and 13.6 (± 27.6) mg Pb/kg soil in treatment (amended) plots

Table S3. Summary information for outcome explanatory models. Information for models with main effects only and for models with main effects + all two-way interactions between them is shown. To make predictions, the model with the lower AIC for each outcome was used (two-way interaction model for all outcomes except runoff nitrate and runoff phosphorus).

Outcome	Model type	AIC	Test for Residual Heterogeneity			Test of Moderators		
			QE	*df*	*p-value*	*QM*	*df*	*p-value*
Soil Organic Carbon	Main Effects Only	686.2	3430.4	231	< 0.0001	650.8	5	< 0.0001
Soil Organic Carbon	With Two-Way Interactions	430.4	2282.6	220	< 0.0001	932.6	16	< 0.0001
Aboveground NPP	Main Effects Only	595.5	2878.1	241	< 0.0001	270.3	4	< 0.0001
Aboveground NPP	With Two-Way Interactions	454.1	1925.6	230	< 0.0001	433.5	15	< 0.0001
Plant Species Diversity	Main Effects Only	-25.0	156.6	92	< 0.0001	14.0	4	0.0072
Plant Species Diversity	With Two-Way Interactions	-34.6	142.5	83	< 0.0001	37.8	13	0.0003
Plant Tissue Nitrogen	Main Effects Only	-78.6	498.5	123	< 0.0001	26.8	4	< 0.0001
Plant Tissue Nitrogen	With Two-Way Interactions	-89.6	392.8	112	< 0.0001	64.1	15	< 0.0001
Soil Lead (Pb)	Main Effects Only	28.1	715.2	83	< 0.0001	50.4	4	< 0.0001
Soil Lead (Pb)	With Two-Way Interactions	25.1	134.4	79	0.0001	64.3	8	< 0.0001
Runoff Quantity	Main Effects Only	281.5	1528.2	74	< 0.0001	55.0	3	< 0.0001
Runoff Quantity	With Two-Way Interactions	173.8	773.4	70	< 0.0001	170.8	7	< 0.0001
Runoff Nitrate	Main Effects Only	131.8	129.0	53	< 0.0001	42.0	3	< 0.0001
Runoff Nitrate	With Two-Way Interactions	139.4	120.9	49	< 0.0001	42.5	7	< 0.0001
Runoff Phosphorus	Main Effects Only	173.8	84.4	62	0.0308	17.7	3	0.0005
Runoff Phosphorus	With Two-Way Interactions	181.0	53.6	58	0.6404	18.1	7	0.0115

Table S4. List of data sources (publications) used to estimate each effect size.

Response Variable	Reference Code List
Soil Organic C	2, 4, 7, 9, 10, 11, 12, 13, 16, 17, 18, 19, 20, 23, 24, 25, 28, 31, 34, 39, 40, 41, 45, 46, 55, 63, 66, 67, 69, 70, 73, 75, 78, 79, 81, 82, 90, 91
Soil Water Holding Capacity	9, 11, 24, 47, 66
Soil Pb	2, 7, 9, 22, 28, 31, 38, 51, 55, 78, 89, 90, 91
Soil CO_2 Emissions (Field)	29, 53, 74
Aboveground NPP	2, 3, 6, 10, 11, 12, 26, 27, 30, 32, 33, 34, 36, 43, 44, 45, 46, 49, 53, 54, 55, 56, 57, 59, 60, 61, 62, 74, 76, 77, 78, 79, 80, 81, 83, 87, 88, 89, 90
Plant Species Diversity	10, 12, 27, 30, 32, 34, 36, 55, 76, 77, 78, 79, 83, 90
Plant Tissue N	2, 6, 31, 46, 50, 52, 55, 59, 60, 68, 69, 78, 86, 88, 90, 91
Plant Tissue Pb	7, 31, 52, 55, 88, 90
Runoff Quantity	1, 2, 3, 16, 21, 24, 37, 54, 58, 64, 71, 72
Runoff Nitrate	1, 2, 21, 37, 54, 65, 72, 84
Runoff P	1, 2, 16, 21, 37, 54, 72, 84
Soil Moisture	16, 20, 24, 35, 37, 38, 45, 47, 53, 92
Soil Respiration (Lab)	4, 8, 9, 10, 11, 12, 13, 24, 34, 41, 45, 46, 78, 82, 92
Soil Microbial Biomass or Abundance	4, 9, 10, 11, 13, 17, 23, 24, 28, 29, 34, 41, 48, 50, 63, 78, 82, 85, 92
Soil Total N	2, 4, 5, 9, 11, 12, 13, 17, 18, 19, 23, 24, 25, 28, 31, 34, 40, 45, 46, 55, 63, 66, 67, 68, 69, 70, 75, 78, 79, 81, 90, 91
Soil Ammonium (NH_4^+)	2, 11, 20, 23, 28, 31, 40, 53, 55, 65, 78, 79, 81, 90, 91
Soil Nitrate (NO_3^-)	2, 4, 11, 20, 23, 28, 31, 40, 42, 53, 55, 65, 78, 79, 80, 81, 90, 91
Soil Extractable P	2, 5, 9, 14, 15, 16, 17, 18, 19, 20, 23, 28, 31, 34, 40, 45, 46, 51, 55, 68, 69, 78, 79, 90, 91
% Cover of Annual Plants	36, 40, 87
% Cover of Grass Plants	6, 49, 59, 60, 87

Reference Codes

1 Aguilar, R., & Loftin, S. R. (1992). Sewage sludge application in semiarid grasslands: Effects on runoff and surface water quality. In C. T. O. Klett (Ed.), *Proceedings of the 36th Annual New Mexico Water Conference: Agencies and Science Working for the Future. WRRI Report No. 265* (pp. 101–111). Las Cruces, NM: New Mexico Water Resources Research Institute.

2 Aguilar, R., Loftin, S. R., Ward, T. J., Stevens, K. A., & Gosz, J. R. (1994). *Sewage sludge application in semiarid grasslands: Effects on vegetation and water quality. Technical completion report.* Las Cruces, NM: New Mexico Water Resources Research Institute.

3 Albaladejo, J., Castillo, V., & Díaz, E. (2000). Soil loss and runoff on semiarid land as amended with urban solid refuse. *Land Degradation and Development*, *11*(4), 363–373. https://doi.org/10.1002/1099-145X(200007/08)11:4<363::AID-LDR399>3.0.CO;2-R

4 Andrés, P., Mateos, E., Tarrasón, D., Cabrera, C., & Figuerola, B. (2011). Effects of digested, composted, and thermally dried sewage sludge on soil microbiota and mesofauna. *Applied Soil Ecology*, *48*(2), 236–242. https://doi.org/10.1016/j.apsoil.2011.03.001

5 Arvas, Ö., Çelebi, Ş. Z., & Yilmaz, I. H. (2011). Effect of sewage sludge and synthetic fertilizer on pH, available N and P in pasture soils in semi-arid area, Turkey. *African Journal of Biotechnology*, *10*(73), 16508–16515. https://doi.org/10.5897/AJB11.110

6 Arvas, Ö., Çelebi, Ş. Z., & Yilmaz, I. H. (2011). The effect of sewage sludge and chemical fertilizer on natural pasture's yield and botanical composition. *Journal of Food, Agriculture and Environment*, *9*(2), 525–530. https://doi.org/https://doi.org/10.1234/4.2011.2159

7 Arvas, Ö., Keskin, B., & Yilmaz, I. H. (2013). Effect of sewage sludge on metal content of grassland soil and herbage in semiarid lands. *Turkish Journal of Agriculture and Forestry*, *37*(2), 179–187. https://doi.org/10.3906/tar-1203-3

8 Barbarick, K. A., Doxtader, K. G., Redente, E. F., & Brobst, R. B. (2004). Biosolids effects on microbial activity in shrubland and grassland soils. *Soil Science*, *169*(3), 176–187. https://doi.org/10.1097/01.ss.0000122525.03492.fe

9 Bastida, F., Moreno, J. L., García, C., & Hernández, T. (2007). Addition of urban waste to semiarid degraded soil: Long-term effect. *Pedosphere*, *17*(5), 557–567. https://doi.org/10.1016/S1002-0160(07)60066-6

10 Bastida, F., Kandeler, E., Moreno, J. L., Ros, M., García, C., & Hernández, T. (2008). Application of fresh and composted organic wastes modifies structure, size and activity of soil microbial community under semiarid climate. *Applied Soil Ecology*, *40*(2), 318–329. https://doi.org/10.1016/j.apsoil.2008.05.007

11 Bastida, F., Pérez-de-Mora, A., Babic, K., Hai, B., Hernández, T., García, C., & Schloter, M. (2009). Role of amendments on N cycling in Mediterranean abandoned semiarid soils. *Applied Soil Ecology*, *41*(2), 195–205. https://doi.org/10.1016/j.apsoil.2008.10.009

12 Bastida, F., Hernández, T., Albaladejo, J., & García, C. (2013). Phylogenetic and functional changes in the microbial community of long-term restored soils under semiarid climate. *Soil Biology and Biochemistry*, *65*, 12–21. https://doi.org/10.1016/j.soilbio.2013.04.022

13 Bastida, F., Selevsek, N., Torres, I. F., Hernández, T., & García, C. (2015). Soil restoration with organic amendments: Linking cellular functionality and ecosystem processes. *Scientific Reports*, *5*(October), 1–12. https://doi.org/10.1038/srep15550

14 Bayley, R. M., Ippolito, J. A., Stromberger, M. E., Barbarick, K. A., & Paschke, M. W. (2008). Water treatment residuals and biosolids coapplications affect semiarid rangeland phosphorus cycling. *Soil Science Society of America Journal*, *72*(3), 711–719. https://doi.org/10.2136/sssaj2007.0109

15 Bell, J. M., Robinson, C. A., & Schwartz, R. C. (2006). Changes in soil properties and enzymatic activities following manure applications to a rangeland. *Rangeland Ecology & Management*, *59*(3), 314–320. https://doi.org/10.2111/05-172R1.1

16 Cabrera, V. E., Stavast, L. J., Baker, T. T., Wood, M. K., Cram, D. S., Flynn, R. P., & Ulery, A. L. (2009). Soil and runoff response to dairy manure application on New Mexico rangeland. *Agriculture, Ecosystems and Environment*, *131*(3–4), 255–262. https://doi.org/10.1016/j.agee.2009.01.022

17 Caravaca, F., Garcia, C., Hernández, M. T., & Roldán, A. (2002). Aggregate stability changes after organic amendment and mycorrhizal inoculation in the afforestation of a semiarid site with Pinus halepensis. *Applied Soil Ecology*, *19*(3), 199–208. https://doi.org/10.1016/S0929-1393(01)00189-5

18 Caravaca, F., Figueroa, D., Azcón-Aguilar, C., Barea, J. M., & Roldán, A. (2003). Medium-term effects of mycorrhizal inoculation and composted municipal waste addition on the establishment of two Mediterranean shrub species under semiarid field conditions. *Agriculture, Ecosystems and Environment*, *97*(1–3), 95–105. https://doi.org/10.1016/S0167-8809(03)00126-9

19 Caravaca, F., Figueroa, D., Alguacil, M. M., & Roldán, A. (2003). Application of composted urban residue enhanced the performance of afforested shrub species in a degraded semiarid land. *Bioresource Technology*, *90*(1), 65–70. https://doi.org/10.1016/S0960-8524(03)00087-7

20 Cellier, A., Gauquelin, T., Baldy, V., & Ballini, C. (2014). Effect of organic amendment on soil fertility and plant nutrients in a post-fire Mediterranean ecosystem. *Plant and Soil*, *376*(1), 211–228. https://doi.org/10.1007/s11104-013-1969-5

21 Crohn, D. M., Chaganti, V. N., & Reddy, N. (2013). Composts as post-fire erosion treatments and their effect on runoff water quality. *Transactions of the ASABE*, *56*(2), 423–435. https://doi.org/10.13031/2013.42692

22 Cuevas, G., Blázquez, R., Martinez, F., & Walter, I. (2000). Composted MSW effects on soil properties and native vegetation in a degraded semiarid shrubland. *Compost Science and Utilization*. https://doi.org/10.1080/1065657X.2000.10702003

23 Dennis, G. L., & Fresquez, P. R. (1989). The soil microbial community in a sewage-sludge-amended semi-arid grassland. *Biology and Fertility of Soils*, *7*(4), 310–317. https://doi.org/10.1007/BF00257825

24 Díaz-Raviña, M., Martín, A., Barreiro, A., Lombao, A., Iglesias, L., Díaz-Fierros, F., & Carballas, T. (2012). Mulching and seeding treatments for post-fire soil stabilisation in NW Spain: Short-term effects and effectiveness. *Geoderma*, *191*, 31–39. https://doi.org/10.1016/j.geoderma.2012.01.003

25 Doni, S., Macci, C., Longo, V., Souid, A., Garcia, C., & Masciandaro, G. (2017). Innovative system for biochemical monitoring of degraded soils restoration. *Catena*, *152*, 173–181. https://doi.org/10.1016/j.catena.2017.01.016

26 Fernández, C., & Vega, J. A. (2014). Efficacy of bark strands and straw mulching after wildfire in NW Spain: Effects on erosion control and vegetation recovery. *Ecological Engineering*, *63*, 50–57. https://doi.org/10.1016/j.ecoleng.2013.12.005

27 Fernández, C., Vega, J. A., Fonturbel, T., Barreiro, A., Lombao, A., Gómez-Rey, M. X., … González-Prieto, S. (2016). Effects of straw mulching on initial post-fire vegetation recovery. *Ecological Engineering*, *95*, 138–142. https://doi.org/10.1016/j.ecoleng.2016.06.046

28 Fresquez, P. R., & Dennis, G. L. (1990). Composition of fungal groups associated with sewage sludge amended grassland soils. *Arid Soil Research and Rehabilitation*, *4*(1), 19–32. https://doi.org/10.1080/15324989009381228

29 Fresquez, P. R., Aldon, E. F., & Dennis, G. L. (1988). *Carbon dioxide evolution from an organically amended Rio Puerco soil. Research Note RM-480. USDA Forest Service Rocky Mountain Forest and Range Experiment Station.* Albuquerque, NM.

30 Fresquez, P. R., Francis, R., & Dennis, G. L. (1990). Soil and vegetation responses to sewage sludge on a degraded semiarid broom snakeweed / blue grama plant community. *Journal of Range Management*, *43*(4), 325–331. https://doi.org/10.2307/3898926

31 Fresquez, P. R., Aguilar, R., Francis, R. E., & Aldon, E. F. (1991). Heavy metal uptake by blue grama growing in a degraded semiarid soil amended with sewage sludge. *Water, Air, and Soil Pollution*, *57*(1), 903–912. https://doi.org/10.1007/BF00282953

32 Gazol, A., Uria-Diez, J., Elustondo, D., Garrigó, J., & Ibáñez, R. (2016). Fertilization triggers 11 years of changes in community assembly in Mediterranean grassland. *Journal of Vegetation Science*, *27*(4), 728–738. https://doi.org/10.1111/jvs.12409

33 Gebremeskel, K., & Pieterse, P. J. (2008). The effect of mulching and fertilising on growth of over-sown grass species in degraded rangeland in north-eastern Ethiopia. *African Journal of Range and Forage Science*, *25*(1), 37–42. https://doi.org/10.2989/AJRFS.2008.25.1.5.383

34 González Polo, M., Kowaljow, E., Castán, E., Sauzet, O., & Mazzarino, M. J. (2015). Persistent effect of organic matter pulse on a sandy soil of semiarid Patagonia. *Biology and Fertility of Soils*, *51*(2), 241–249. https://doi.org/10.1007/s00374-014-0961-4

35 Hanke, W., Gröngröft, A., Jürgens, N., & Schmiedel, U. (2011). Rehabilitation of arid rangelands: Intensifying water pulses from low-intensity winter rainfall. *Journal of Arid Environments*, *75*(2), 185–193. https://doi.org/10.1016/j.jaridenv.2010.09.002

36 Hanke, W., Wesuls, D., Münchberger, W., & Schmiedel, U. (2015). Tradeoffs in the rehabilitation of a succulent Karoo rangeland. *Land Degradation & Development*, *26*(8), 833–842. https://doi.org/10.1002/ldr.2224

37 Harris-Pierce, R. L., Redente, E. F., & Barbarick, K. A. (1995). Sewage sludge application effects on runoff water quality in a semiarid grassland. *Journal of Environmental Quality*, *24*(1), 112–115. https://doi.org/10.2134/jeq1995.00472425002400010016x

38 Illera, V., Walter, I., Souza, P., & Cala, V. (2000). Short-term effects of biosolid and municipal solid waste applications on heavy metals distribution in a degraded soil under a semi-arid environment. *Science of the Total Environment*, *255*(1–3), 29–44. https://doi.org/10.1016/S0048-9697(00)00444-7

39 Ippolito, J. A., Barbarick, K. A., & Brobst, R. B. (2009). Fate of biosolids Cu and Zn in a semi-arid grassland. *Agriculture, Ecosystems and Environment*, *131*(3–4), 325–332. https://doi.org/10.1016/j.agee.2009.02.013

40 Ippolito, J. A., Barbarick, K. A., Paschke, M. W., & Brobst, R. B. (2010). Infrequent composted biosolids applications affect semi-arid grassland soils and vegetation. *Journal of Environmental Management*, *91*(5), 1123–1130. https://doi.org/10.1016/j.jenvman.2010.01.004

41 Jorge-Mardomingo, I., Soler-Rovira, P., Casermeiro, M. Á., de la Cruz, M. T., & Polo, A. (2013). Seasonal changes in microbial activity in a semiarid soil after application of a high dose of different organic amendments. *Geoderma*, *206*, 40–48. https://doi.org/10.1016/j.geoderma.2013.04.025

42 Jurado-Guerra, P., Wester, D. B., & Fish, E. B. (2006). Soil nitrate nitrogen dynamics after biosolids application in tobosagrass desert grassland. *Journal of Environmental Quality*, *35*(2), 641–650. https://doi.org/10.2134/jeq2005.0062

43 Jurado-Guerra, P., Luna-Luna, M., Flores-Ancira, E., & Saucedo-Teran, R. (2013). Residual effects of biosolids application on forage production of semiarid grassland in Jalisco, Mexico. *Applied and Environmental Soil Science*, Article ID 835960. https://doi.org/10.1155/2013/835960

44 Jurado, P., & Wester, D. B. (2001). Effects of biosolids on tobosagrass growth in the Chihuahuan Desert. *Journal of Range Management*, *54*(1), 89–95. https://doi.org/10.2307/4003534

45 Kowaljow, E., & Mazzarino, M. J. (2007). Soil restoration in semiarid Patagonia: Chemical and biological response to different compost quality. *Soil Biology and Biochemistry*, *39*(7), 1580–1588. https://doi.org/10.1016/j.soilbio.2007.01.008

46 Kowaljow, E., Mazzarino, M. J., Satti, P., & Jiménez-Rodríguez, C. (2010). Organic and inorganic fertilizer effects on a degraded Patagonian rangeland. *Plant and Soil*, *332*, 135–145. https://doi.org/10.1007/s11104-009-0279-4

47 Kowaljow, E., Gonzalez-Polo, M., & Mazzarino, M. J. (2017). Understanding compost effects on water availability in a degraded sandy soil of Patagonia. *Environmental Earth Sciences*, *76*(6), 1–10. https://doi.org/10.1007/s12665-017-6573-1

48 Labidi, S., Nasr, H., Zouaghi, M., & Wallander, H. (2007). Effects of compost addition on extra-radical growth of arbuscular mycorrhizal fungi in Acacia tortilis ssp. raddiana savanna in a pre-Saharan area. *Applied Soil Ecology*, *35*(1), 184–192. https://doi.org/10.1016/j.apsoil.2006.04.009

49 Larchevêque, M., Montès, N., Baldy, V., & Dupouyet, S. (2005). Vegetation dynamics after compost amendment in a Mediterranean post-fire ecosystem. *Agriculture, Ecosystems and Environment*, *110*(3–4), 241–248. https://doi.org/10.1016/j.agee.2005.04.006

50 Larchevêque, M., Baldy, V., Korboulewsky, N., Ormeño, E., & Fernandez, C. (2005). Compost effect on bacterial and fungal colonization of kermes oak leaf litter in a terrestrial Mediterranean ecosystem. *Applied Soil Ecology*, *30*(2), 79–89. https://doi.org/10.1016/j.apsoil.2005.02.010

51 Larchevêque, M., Baldy, V., Montès, N., Fernandez, C., Bonin, G., & Ballini, C. (2006). Short-term effects of sewage-sludge compost on a degraded Mediterranean soil. *Soil Science Society of America Journal*, *70*(4), 1178–1188. https://doi.org/10.2136/sssaj2005.0115

52 Larchevêque, M., Ballini, C., Baldy, V., Korboulewsky, N., Ormeño, E., & Montès, N. (2010). Restoration of a Mediterranean postfire shrubland: Plant functional responses to organic soil amendment. *Restoration Ecology*, *18*(5), 729–741. https://doi.org/10.1111/j.1526-100X.2008.00512.x

53 Louro, A., Cárdenas, L. M., García, M. I., & Báez, D. (2016). Greenhouse gas fluxes from a grazed grassland soil after slurry injections and mineral fertilizer applications under the Atlantic climatic conditions of NW Spain. *Science of the Total Environment*, *573*, 258–269. https://doi.org/10.1016/j.scitotenv.2016.08.092

54 Martínez, F., Casermeiro, M. A., Morales, D., Cuevas, G., & Walter, I. (2003). Effects on run-off water quantity and quality of urban organic wastes applied in a degraded semi-arid ecosystem. *Science of the Total Environment*, *305*, 13–21. https://doi.org/10.1016/S0048-9697(02)00472-2

55 Martínez, F., Cuevas, G., Calvo, R., & Walter, I. (2003). Biowaste effects on soil and native plants in a semiarid ecosystem. *Journal of Environment Quality*, *32*(2), 472–479. https://doi.org/10.2134/jeq2003.4720

56 McFarland, M. J., Vasquez, I. R., Vutran, M., Schmitz, M., & Brobst, R. B. (2010). Use of Biosolids to Enhance Rangeland Forage Quality. *Water Environment Research*, *82*(5), 455–461. https://doi.org/10.2175/106143009X12529484815872

57 McKell, C. M., Brown, V. W., Adolph, R. H., & Duncan, C. (1970). Fertilization of annual rangeland with chicken manure. *Journal of Range Management*, *23*(5), 336–340. https://doi.org/10.2307/3896162

58 Moffet, C. A., Zartman, R. E., Wester, D. B., & Sosebee, R. E. (2005). Surface biosolids application: Effects on infiltration, erosion, and soil organic carbon in Chihuahuan Desert grasslands and shrublands. *Journal of Environmental Quality*, *34*(1), 299–311. https://doi.org/10.2134/jeq2005.0299

59 Mut, H., Ayan, I., Basaran, U., Onal-Asci, O., & Acar, Z. (2010). The effects of sheep manure application time and rates on yield and botanical composition of secondary succession rangeland. *African Journal of Biotechnology*, *9*(23), 3388–3395. https://doi.org/10.5897/AJB10.362

60 Mut, H., Ayan, I., Acar, Z., Basaran, U., & Onal-Asci, Ö. (2010). The effect of different improvement methods on pasture yield and quality of hay obtained from the abandoned rangeland. *Turkish Journal of Field Crops*, *15*(2), 198–203.

61 Navas, A., Machín, J., & Navas, B. (1999). Use of biosolids to restore the natural vegetation cover on degraded soils in the badlands of Zaragoza (NE Spain). *Bioresource Technology*, *69*(3), 199–205. https://doi.org/10.1016/S0960-8524(99)00003-6

62 Newman, R. F., Krzic, M., & Wallace, B. M. (2014). Differing effects of biosolids on native plants in grasslands of southern British Columbia. *Journal of Environment Quality*, *43*(5), 1672. https://doi.org/10.2134/jeq2014.01.0013

63 Nicolás, C., Kennedy, J. N., Hernández, T., García, C., & Six, J. (2014). Soil aggregation in a semiarid soil amended with composted and non-composted sewage sludge-A field experiment. *Geoderma*, *219–220*, 24–31. https://doi.org/10.1016/j.geoderma.2013.12.017

64 Ojeda, G., Alcañiz, J. M., & Ortiz, O. (2003). Runoff and losses by erosion in soils amended with sewage sludge. *Land Degradation & Development*, *14*(6), 563–573. https://doi.org/10.1002/ldr.580

65 Ojeda, G., Tarrasón, D., Ortiz, O., & Alcañiz, J. M. (2006). Nitrogen losses in runoff waters from a loamy soil treated with sewage sludge. *Agriculture, Ecosystems and Environment*, *117*(1), 49–56. https://doi.org/10.1016/j.agee.2006.02.017

66 Ojeda, G., Perfect, E., Alcañiz, J. M., & Ortiz, O. (2006). Fractal analysis of soil water hysteresis as influenced by sewage sludge application. *Geoderma*, *134*(3–4), 386–401. https://doi.org/10.1016/j.geoderma.2006.03.011

67 Ojeda, G., Alcañiz, J. M., & Le Bissonnais, Y. (2008). Differences in aggregate stability due to various sewage sludge treatments on a Mediterranean calcareous soil. *Agriculture, Ecosystems and Environment*, *125*(1–4), 48–56. https://doi.org/10.1016/j.agee.2007.11.005

68 Olivier, R., Lavoir, A. V., Ormeño, E., Mouillot, F., Greff, S., Lecareux, C., … Fernandez, C. (2011). Compost spreading in Mediterranean shrubland indirectly increases biogenic emissions by promoting growth of VOC-emitting plant parts. *Atmospheric Environment*, *45*, 3631–3639. https://doi.org/10.1016/j.atmosenv.2011.03.060

69 Ormeño, E., Olivier, R., Mévy, J. P., Baldy, V., & Fernandez, C. (2009). Compost may affect volatile and semi-volatile plant emissions through nitrogen supply and chlorophyll fluorescence. *Chemosphere*, *77*(1), 94–104. https://doi.org/10.1016/j.chemosphere.2009.05.014

70 Owen, J. J., Parton, W. J., & Silver, W. L. (2015). Long-term impacts of manure amendments on carbon and greenhouse gas dynamics of rangelands. *Global Change Biology*, *21*(12), 4533–4547. https://doi.org/10.1111/gcb.13044

71 Ros, M., Garcia, C., & Hernandez, T. (2001). The use of urban organic wastes in the control of erosion in a semiarid Mediterranean soil. *Soil Use and Management*, *17*(4), 292–293. https://doi.org/10.1111/j.1475-2743.2001.tb00041.x

72 Rostagno, C. M., & Sosebee, R. E. (2001). Biosolids application in the Chihuahuan desert: Effects on runoff water quality. *Journal of Environmental Quality*, *30*(1), 160–170. https://doi.org/10.2134/jeq2001.301160x

73 Rostagno, C. M., & Sosebee, R. B. (2001). Surface application of biosolids in the Chihuahuan Desert: Effects on soil physical properties. *Arid Land Research and Management*, *15*(3), 233–244. https://doi.org/10.1080/15324980152119793

74 Ryals, R., & Silver, W. L. (2013). Effects of organic matter amendments on net primary productivity and greenhouse gas emissions in annual grasslands. *Ecological Applications*, *23*(1), 46–59. https://doi.org/10.1890/12-0620.1

75 Ryals, R., Kaiser, M., Torn, M. S., Berhe, A. A., & Silver, W. L. (2014). Impacts of organic matter amendments on carbon and nitrogen dynamics in grassland soils. *Soil Biology and Biochemistry*, *68*, 52–61. https://doi.org/10.1016/j.soilbio.2013.09.011

76 Ryals, R., Eviner, V. T., Suding, K. N., & Silver, W. L. (2016). Grassland compost amendments increase plant production without changing plant communities. *Ecosphere*, *7*(3), Article e01270. https://doi.org/10.1002/ecs2.1270

77 Stavast, L. J., Baker, T. T., Ulery, A. L., Flynn, R. P., Wood, M. K., & Cram, D. S. (2005). New Mexico blue grama rangeland response to dairy manure application. *Rangeland Ecology & Management*, *58*(4), 423–429.

78 Sullivan, T. S., Stromberger, M. E., Paschke, M. W., & Ippolito, J. A. (2006). Long-term impacts of infrequent biosolids applications on chemical and microbial properties of a semi-arid rangeland soil. *Biology and Fertility of Soils*, *42*(3), 258–266. https://doi.org/10.1007/s00374-005-0023-z

79 Sullivan, T. S., Stromberger, M. E., & Paschke, M. W. (2006). Parallel shifts in plant and soil microbial communities in response to biosolids in a semi-arid grassland. *Soil Biology and Biochemistry*, *38*(3), 449–459. https://doi.org/10.1016/j.soilbio.2005.05.018

80 Tarrasón, D., Ortiz, O., & Alcañiz, J. M. (2007). A multi-criteria evaluation of organic amendments used to transform an unproductive shrubland into a Mediterranean dehesa. *Journal of Environmental Management*, *82*(4), 446–456. https://doi.org/10.1016/j.jenvman.2006.01.002

81 Tarrasón, D., Ojeda, G., Ortiz, O., & Alcañiz, J. M. (2008). Differences on nitrogen availability in a soil amended with fresh, composted and thermally-dried sewage sludge. *Bioresource Technology*, *99*(2), 252–259. https://doi.org/10.1016/j.biortech.2006.12.023

82 Tarrasón, D., Ojeda, G., Ortiz, O., & Alcañiz, J. M. (2010). Effects of different types of sludge on soil microbial properties: A field experiment on degraded Mediterranean soils. *Pedosphere*, *20*(6), 681–691. https://doi.org/10.1016/S1002-0160(10)60058-6

83 Tarrasón, D., Ojeda, G., Ortiz, O., & Alcañiz, J. M. (2014). Can organic amendments be useful in transforming a Mediterranean shrubland into a dehesa? *Restoration Ecology*, *22*(4), 486–494. https://doi.org/10.1111/rec.12092

84 Tejada, M., & Gonzalez, J. L. (2008). Influence of two organic amendments on the soil physical properties, soil losses, sediments and runoff water quality. *Geoderma*, *145*, 325–334. https://doi.org/10.1016/j.geoderma.2008.03.020

85 Torres, I. F., Bastida, F., Hernández, T., Albaladejo, J., & García, C. (2015). Enzyme activity, microbial biomass and community structure in a long-term restored soil under semi-arid conditions. *Soil Research*, *53*(5), 553–560. https://doi.org/10.1071/SR14297

86 Valdecantos, A., Cortina, J., & Vallejo, V. R. (2011). Differential field response of two Mediterranean tree species to inputs of sewage sludge at the seedling stage. *Ecological Engineering*, *37*(9), 1350–1359. https://doi.org/10.1016/j.ecoleng.2011.03.017

87 Wallace, B. M., Krzic, M., Newman, R. F., Forge, T. A., Broersma, K., & Neilsen, G. (2016). Soil aggregate dynamics and plant community response after biosolids application in a semiarid grassland. *Journal of Environmental Quality*, *45*(5), 1663–1671. https://doi.org/10.2134/jeq2016.01.0030

88 Walter, I., & Calvo, R. (2009). Biomass production and development of native vegetation following biowaste amendment of a degraded, semi-arid soil. *Arid Land Research and Management*, *23*(4), 297–310. https://doi.org/10.1080/15324980903231827

89 Walter, I., Cuevas, G., García, S., & Martínez, F. (2000). Biosolid effects on soil and native plant production in a degraded semiarid ecosystem in central Spain. *Waste Management and Research*, *18*(3), 259–263. https://doi.org/10.1034/j.1399-3070.2000.00111.x

90 Walter, I., Martínez, F., & Cuevas, G. (2006). Plant and soil responses to the application of composted MSW in a degraded, semiarid shrubland in central Spain. *Compost Science and Utilization*, *14*(2), 147–154. https://doi.org/10.1080/1065657X.2006.10702276

91 White, C. S., Loftin, S. R., & Aguilar, R. (1997). Application of biosolids to degraded semiarid rangeland: Nine-year responses. *Journal of Environmental Quality*, *26*(6), 1663–1671. https://doi.org/10.2134/jeq1997.00472425002600060029x

92 Whitford, W. G., Aldon, E. F., Freckman, D. W., Steinberger, Y., & Parker, L. W. (1989). Effects of organic amendments on soil biota on a degraded rangeland. *Journal of Range Management*, *42*(1), 56–60. https://doi.org/10.2307/3899659

Table S5. Predicted effects of amendment amount + amendment N concentration scenarios across outcomes. In predictive models, time between amendment application and measurement was set to three years; longer-term outcomes are of interest, but we chose to model scenarios that were well within the range of available data (Table S1). To determine which scenarios best "maximized benefits" and "minimized harms", predicted effect sizes whose 95% confidence intervals (CIs) *excluded* 0 were the most important criteria, with predicted effect sizes whose 95% CIs *included* 0 then also considered in order to gain better resolution. According to this framework, scenarios that maximized benefits are highlighted in blue and scenarios that minimized harms are highlighted in orange. Importantly, our concept of "benefits" and "harms" here refers only to the direction of the effect sizes (and the assumed societal consequences of these directions, Table 3); it does not consider effect size magnitudes relative to external benefit or harm definitions (e.g., regulatory standards, ranchers' perceptions of meaningful changes) because such definitions are likely to vary across contexts. Outcomes are abbreviated as follows: soil organic C (soilC), aboveground net primary productivity (ANPP), plant species diversity (spdiv), plant tissue N (plantN), soil Pb (soilPb), runoff quantity (runoffquant), runoff nitrate (runoffN), and runoff P (runoffP).

**Amendment N concentration (%)**	**Amount of amendment applied (Mg / ha)**	**Outcomes where effect direction indicates assumed societal *benefits* and CI excludes 0 (*more certain*)**	**Outcomes where effect direction indicates assumed societal *benefits* and CI includes 0 (*less certain*)**	**Outcomes where effect direction indicates assumed societal *harms* and CI includes 0 (*less certain*)**	**Outcomes where effect direction indicates assumed societal *harms* and CI excludes 0 (*more certain*)**
1.2	10	plantN	ANPP, spdiv, runoffquant	runoffN, runoffP	soilC, soilPb
1.2	20	plantN	ANPP, spdiv, runoffquant	soilC, runoffN	runoffP, soilPb
1.2	30	plantN, runoffquant	soilC, ANPP, spdiv	runoffN	runoffP, soilPb
1.2	40	plantN, runoffquant	soilC, ANPP, spdiv		runoffN, runoffP, soilPb
1.2	50	soilC, ANPP, plantN, runoffquant	spdiv		runoffN, runoffP, soilPb
1.8	10	plantN	ANPP, spdiv, runoffquant	soilC, runoffN, runoffP, soilPb	
1.8	20	plantN	soilC, ANPP, spdiv, runoffquant		runoffN, runoffP, soilPb
1.8	30	plantN, runoffquant	soilC, ANPP, spdiv		runoffN, runoffP, soilPb
1.8	40	soilC, ANPP, plantN, runoffquant	spdiv		runoffN, runoffP, soilPb
1.8	50	soilC, ANPP, plantN, runoffquant	spdiv		runoffN, runoffP, soilPb
2.4	10	plantN	ANPP, spdiv, runoffquant	soilC, runoffN, soilPb	runoffP
2.4	20	plantN, runoffquant	soilC, ANPP	spdiv, soilPb	runoffN, runoffP

2.4	30	soilC, ANPP, plantN, runoffquant		spdiv	runoffN, runoffP, soilPb
2.4	40	soilC, ANPP, plantN, runoffquant		spdiv	runoffN, runoffP, soilPb
2.4	50	soilC, ANPP, plantN, runoffquant		spdiv	runoffN, runoffP, soilPb
3.0	10	plantN, runoffquant	soilC, ANPP	spdiv, soilPb	runoffN, runoffP
3.0	20	soilC, plantN, runoffquant	ANPP	spdiv, soilPb	runoffN, runoffP
3.0	30	soilC, ANPP, plantN, runoffquant		spdiv, soilPb	runoffN, runoffP
3.0	40	soilC, ANPP, plantN, runoffquant		spdiv, soilPb	runoffN, runoffP
3.0	50	soilC, ANPP, plantN, runoffquant		spdiv, soilPb	runoffN, runoffP
3.6	10	runoffquant	soilC, ANPP, plantN	spdiv, soilPb	runoffN, runoffP
3.6	20	soilC, ANPP, plantN, runoffquant		spdiv, soilPb	runoffN, runoffP
3.6	30	soilC, ANPP, plantN, runoffquant		spdiv, soilPb	runoffN, runoffP
3.6	40	soilC, ANPP, plantN, runoffquant		soilPb	spdiv, runoffN, runoffP
3.6	50	soilC, ANPP, plantN, runoffquant		soilPb	spdiv, runoffN, runoffP

Figure S1. Interaction between amount of amendment applied and amendment N concentration in explanatory models for four outcomes: (a) plant species diversity (importance = 0.9997), (b) plant tissue N (importance = 0.9602), (c) soil organic C (importance = 1), and (d) runoff quantity (importance = 1). Note log-log axes. Points represent observations (unique experiment + measurement date + amendment type + amount applied combinations) and are proportional to the inverse of effect size standard errors, such that larger points indicate more precise observations. Points are color-coded by amendment N concentration, with orange points indicating amendments with < 2.4% N and blue points indicating amendments with ≥ 2.4% N. Lines represent model predictions, with all other variables in the model set to their means.

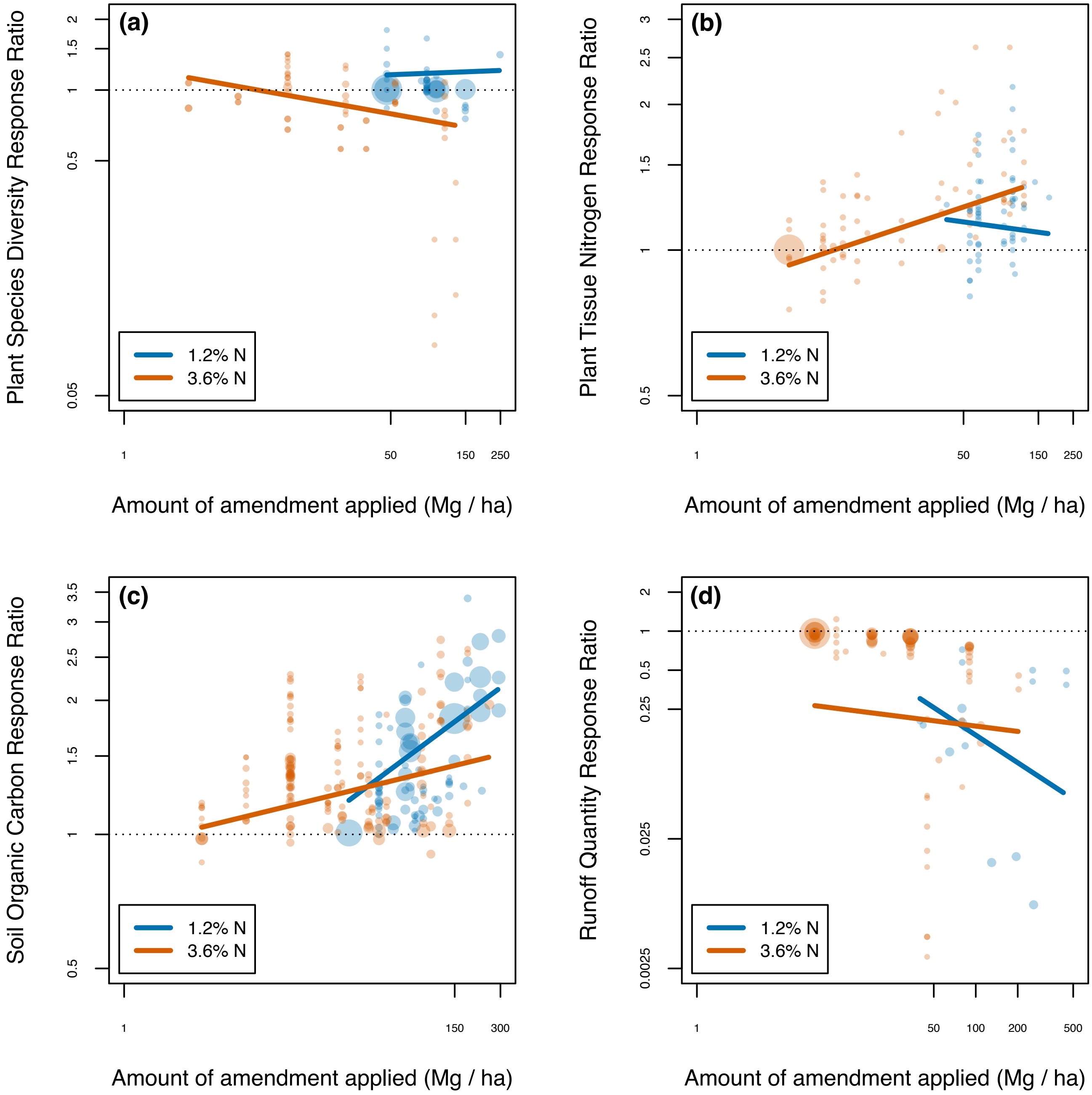

Figure S2. Predicted effect sizes (log response ratios) for outcomes under three amendment amount + amendment N concentration scenarios: Spider plots. An alternative depiction of the scenario predictions shown in Figure 6. The red line shows the (mean) predicted effect size, the dark gray shading indicates the 95% confidence interval around that mean, and the black line indicates a log response ratio of 0 (no effect). Here, outcomes for which a positive effect size indicates assumed societal benefits are shown in *blue text*; for these outcomes, the red outside the black line indicates an assumed benefit. Outcomes for which a positive effect size indicates assumed societal harms are shown in *orange text*; for these outcomes, the red outside the black line indicates an assumed harm. Each plot shows a scenario.

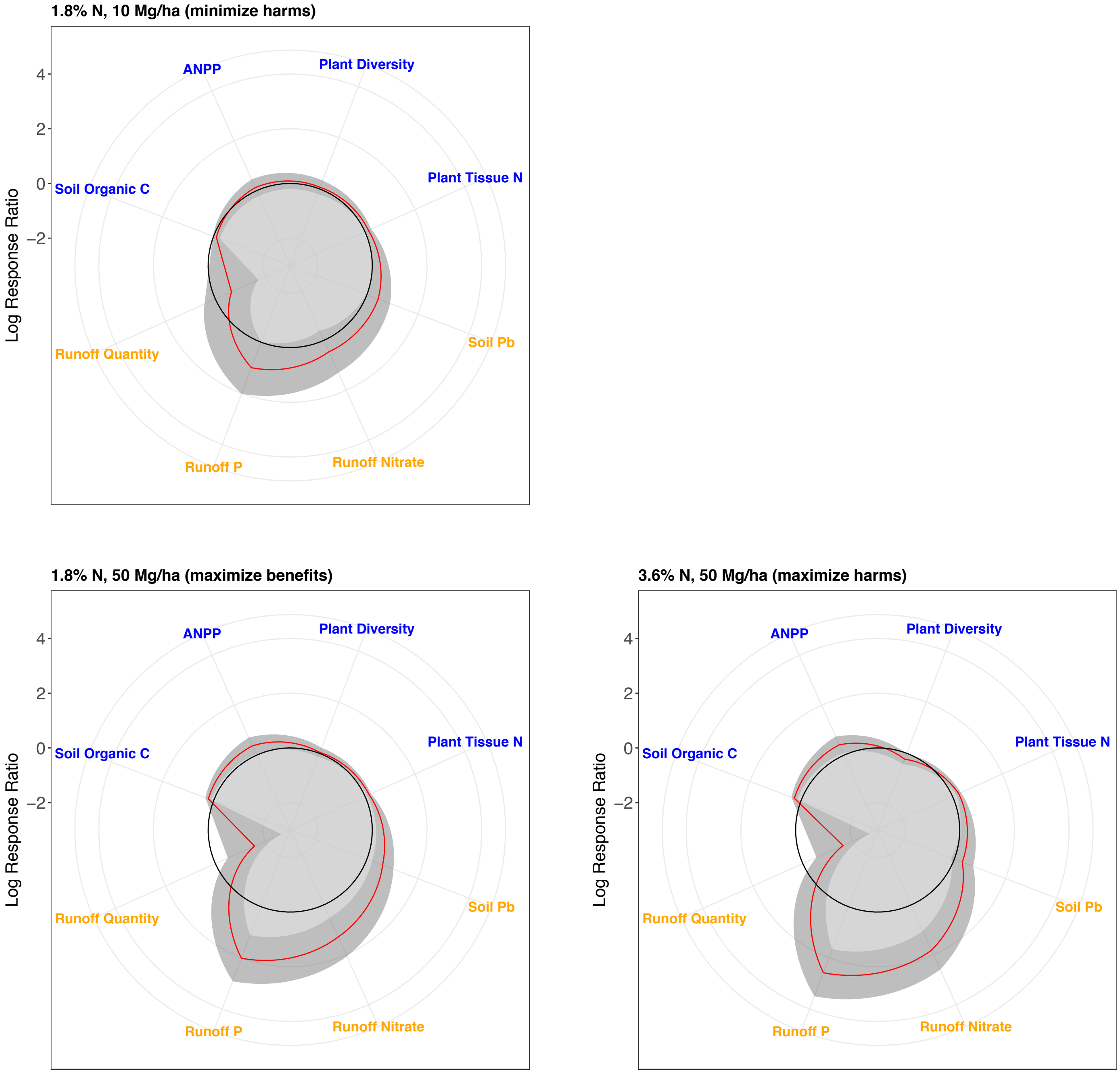

Supplement: Supplementary file 1 [file GCB-25-1152-s001.pdf]
